# Steroid receptor coactivator 3 (*SRC-3*/*AIB1*) is enriched and functional in mouse and human Tregs

**DOI:** 10.1038/s41598-021-82945-3

**Published:** 2021-02-09

**Authors:** Bryan C. Nikolai, Prashi Jain, David L. Cardenas, Brian York, Qin Feng, Neil J. McKenna, Subhamoy Dasgupta, David M. Lonard, Bert W. O’Malley

**Affiliations:** 1 Department of Molecular and Cellular Biology, Baylor College of Medicine, One Baylor Plaza, Houston, TX 77030 USA; 2 Center for Nuclear Receptors and Cell Signaling, University of Houston, Houston, TX 77204 USA; 3 Department of Oncology, Roswell Park Comprehensive Cancer Center, Buffalo, NY 14263 USA; 4 Laboratory of Molecular Regulation, Baylor College of Medicine, Houston, TX 77030 USA

**Keywords:** Cell biology, Chemical biology, Immunology

## Abstract

A subset of CD4 + lymphocytes, regulatory T cells (Tregs), are necessary for central tolerance and function as suppressors of autoimmunity against self-antigens. The SRC-3 coactivator is an oncogene in multiple cancers and is capable of potentiating numerous transcription factors in a wide variety of cell types. *Src-3* knockout mice display broad lymphoproliferation and hypersensitivity to systemic inflammation. Using publicly available bioinformatics data and directed cellular approaches, we show that SRC-3 also is highly enriched in Tregs in mice and humans. Human Tregs lose phenotypic characteristics when SRC-3 is depleted or pharmacologically inhibited, including failure of induction from resting T cells and loss of the ability to suppress proliferation of stimulated T cells. These data support a model for SRC-3 as a coactivator that actively participates in protection from autoimmunity and may support immune evasion of cancers by contributing to the biology of Tregs.

## Introduction

SRC-3 functions as a transcriptional coactivator for steroid hormone receptors including the estrogen, progesterone and numerous other nuclear receptors, but also functions as a primary coactivator for many other DNA-binding transcription factors including E2F, NFκB, and members of the ETS family^1–4^. SRC-3 can be extensively modified by post-translational modifications (PTMs), including phosphorylation, methylation, acetylation, and ubiquitination that regulate its function and affinity for transcription factors and coactivator protein complexes^5,6^. These combinatorial PTMs are reflective of the environment of hormones, cytokines, and environment to which cells are exposed and the state of their respective signaling pathways. Since SRC-3 can assimilate diverse cellular cues in this way, it is thought to function as a nutrient sensor and signaling integrator to drive diverse gene expression patterns that reflect the needs of the cell.

It is well established that breast cancer is a heterogeneous disease, both in terms of tumor subtypes present in patient populations and the milieu of cell types present within individual tumors. An important component of the tumor microenvironment is infiltrating immune cells that can serve various roles in controlling tumor growth or providing local immune suppression, effectively protecting the tumor from destruction by the immune system. Various suppressive immune cell types from myeloid or lymphoid origins are known to block anti-tumor immunity, including myeloid-derived suppressor cells (MDSCs), tumor associated macrophages (TAMs), and regulatory T cells (Tregs). The latter cell type is of particular interest since Treg infiltration has been experimentally proven in breast tumor biopsies and correlates with poor prognosis and reduced breast cancer patient survival^7,8^.

Tregs are critical to prevent autoimmunity in peripheral tissues where they can suppress local immunity and contribute to self-tolerance. A hallmark of Tregs is their intracellular expression of the forkhead box transcription factor 3 (Foxp3), which is critical for Treg suppressive immune function. Mice with mutations or deletions of *FOXP3* develop lethal autoimmunity and humans with mutations in *FOXP3* develop a rare disease called immunodysregulation polyendocrinopathy enteropathy X-linked (IPEX) syndrome. Autoimmunity, thrombocytopenia, alopecia, erythematous plaques and general dermatitis characterize these diseases. In severe cases, neonates fail to thrive and require bone marrow transplantation. Conversely, Treg function can be hijacked by tumors or viruses to evade immune clearance. Regulation of the *FOXP3* gene is thus a critical determinant for immune self-tolerance, and factors controlling *FOXP3* transcription may be attractive targets to modulate Treg activity.

## Results

The role of SRC-3 in the immune system is not well understood at present, despite publications showing high SRC-3 expression in leukocytes. We have found that knockout of the *Src-3* gene in mice results in phenotypes that include broad lymphoproliferation and hypersensitivity to acute inflammation^9,10^. Our aged *Src-3* null mice develop B-cell lymphomas and display elevated numbers of B cells and T cells in lymph nodes, spleen, and bone marrow^9^. Additionally, *Src-3* knockout mice challenged with LPS experience endotoxic shock and are unable to defend body temperature or dampen inflammatory cytokines including TNFα, IL-6, and IL-1β^10^. To confirm that these immune phenotypes have not drifted from our previous reports, we performed comprehensive immune phenotyping of spleens from our *Src-3* knockout mice and age-matched littermates. We used an antibody panel established by the Cell Cytometry and Sorting Core at Baylor College of Medicine in collaboration with the International Mouse Phenotyping Consortium (See Supplementary Fig. 1A online). Metadata for mouse age, weight, body temperature, blood glucose, tibia length, splenocytes number and viability, and the number of FACS events were collected (See Supplementary Fig. 1B online). No overt inguinal lymphomas were observed by gross inspection in any of the knockout or wild-type mice. Additionally, and consistent with general dwarfism observed in the *Src-3* knockout mice^11,12^ spleens were not enlarged relative to littermates (data not shown). We observe broad lymphoproliferation of T cells and B cells (See Supplementary Fig. 1C and Supplementary File 1 online), while natural killer (NK) cells and myeloid lineages were unchanged or slightly decreased (See Supplementary Fig. 1D and Supplementary File 1 online). Collectively, these results corroborate previous reports and suggest that loss of SRC-3 conveys a stable immune phenotype that is sensitive to lymphocyte-mediated inflammatory stress.

**Figure 1:**
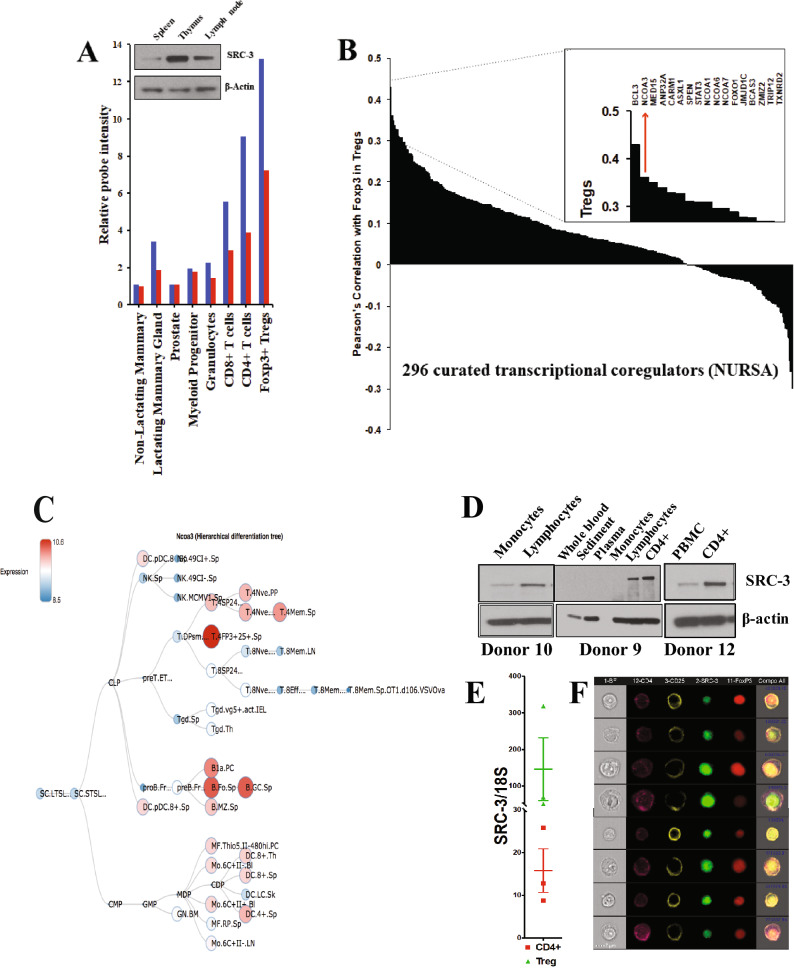
Steroid Receptor Coactivator 3 (SRC-3/NCOA3) is highly expressed in Tregs. (**A**) *Ncoa3/Src-3* probe intensity in various tissues from BioGPS database (Gel image cropped from original blot submitted as supplemental figures) and the blue and red bars each represent expression values from two different probes from the microarray used in the BioGPS database. (**B**) Correlation of expression between NURSA coactivators and *Foxp3* in Tregs using ImmunoNavigator. (**C**) Hematopoietic lineage map of *Ncoa3/Src-3* expression showing highest levels in CD4^+^CD25^+^FOXP3^+^ Tregs in the spleen. (**D**) Fractionation of human blood shows elevated SRC-3 protein levels in CD4 + lymphocytes (Gel image cropped from original blot submitted as supplemental figures). (**E**) Enriched transcript levels of *SRC-3* in freshly isolated Tregs in humans. (**F**) Imaging cytometry shows SRC-3 protein expression in CD4^+^CD25^+^FOXP3^+^ human Tregs.

We noted high expression of SRC-3 protein in the thymus of healthy C57BL6/J mice (Fig. 1A, inset) and took an explorative bioinformatics approach to identify the SRC-3 immune compartment. Query of microarray data in the BioGPS web portal shows that the *Src-3* transcript is highly expressed in T cells and B cells, and enriched in regulatory T cells (Tregs) (Fig. 1A). Similarly, microarray and more recent RNA sequencing data available through the Immune Genome project (ImmGen)^13–15^ indicates that the *Src-3* transcript is highly expressed in regulatory T cells (See Supplementary Fig. 1E online). Next, we sought to use an orthogonal approach and assembled a curated list of 297 transcriptional coregulators from the Nuclear Receptor Signaling Atlas (NURSA)^16^. This coregulator gene list was parsed against *Foxp3* transcript expression using the gene set enrichment analysis (GSEA) function in ImmunoNavigator^17^. Of the 297 best-curated transcriptional coregulators from NURSA, *Ncoa3* (*Src-3*) expression is the second-most correlated coregulator with *Foxp3* expression (Fig. 1B). Visualization of *Src-3* expression using hematopoietic lineage mapping using BloodSpot^18^ further demonstrates Treg enrichment and suggests that the *Src-3* transcript is higher in Tregs than any other hematopoietic cell type (Fig. 1C). Taken together, a breadth of bioinformatics data supports high expression of SRC-3 in Tregs.

Since *Src-3* transcript is highly abundant in mouse Tregs and considering that Tregs have important functions in human disease, we tested whether SRC-3 protein and transcript are likewise elevated in human cells obtained from healthy blood donors. Similar to our observations in mouse tissues, human SRC-3 is enriched in lymphocytes relative to monocytes and bulk peripheral blood mononuclear cells (PBMC) (Fig. 1D). We next prepared matched samples of bulk CD4 cells and Tregs from donor blood and directly compared transcript levels of *SRC-3*. Indeed, in all three donors we tested, relative *SRC-3* expression is significantly higher in Tregs than bulk CD4 cell preparations (Fig. 1E). Analysis of Tregs from one of these donors using fixation/permeabilization fluor-conjugated antibody staining and imaging flow cytometry confirmed the nuclear expression of SRC-3 in FOXP3-expressing Tregs (Fig. 1F). In summary, we find that SRC-3 is significantly enriched in Tregs in mice and humans.

**Figure 2:**
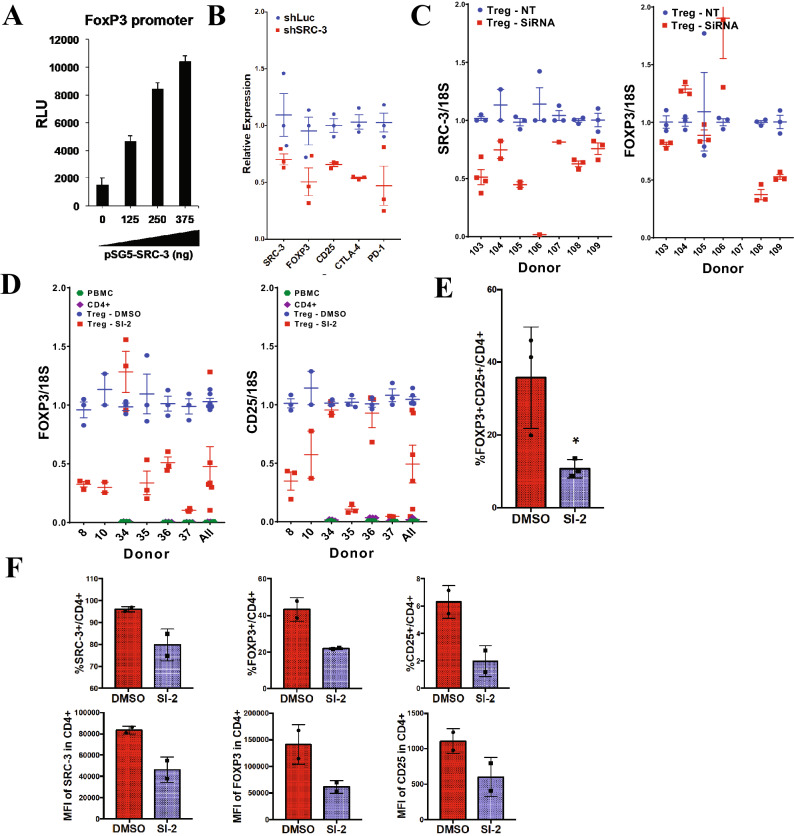
SRC-3 knockdown or inhibition in human Tregs reduces FOXP3 and CD25 levels. (**A**) SRC-3 transactivates FOXP3 promoter in transiently transfection assay. (**B**) RNAi knockdown of SRC-3 in Tregs decreases transcript levels of *FOXP3, CD25, CTLA-4, and PD-1*. (**C**) SRC-3 knockdown in Tregs decreases transcript levels of *SRC-3*, *FOXP3* and *CD25* in multiple human donors. (**D**) Inhibition of SRC-3 with SI-2 (100 nM for 12 h) in Tregs decreases transcript levels of *FOXP3* and *CD25* in multiple human donors. (**E**) SRC-3 inhibition reduces protein expression of FOXP3 and CD25 in Tregs from multiple human donors measured using traditional flow cytometry and (**F**) imaging cytometry.

Since transcriptional regulation in Tregs is critical for cell type lineation, identity, and function, we considered that the observed enrichment of the SRC-3 transcriptional coactivator could influence expression of typical Treg marker genes. A luciferase reporter construct including the promoter region of *Foxp3* is transactivated by SRC-3 in a dose-dependent manner (Fig. 2A). Knockdown of SRC-3 using lentiviral shRNA, although relatively inefficient, decreases Treg marker gene transcripts *FOXP3* and *IL2RA* (*CD25*) and checkpoint receptor transcripts *PD-1* and *CTLA-4* (Fig. 2B). Our initial attempts to completely knockdown SRC-3 in these cells using shRNA were met with less success and low cell viability. As has been previously reported^19–21^ primary human cells (PBMCs) are difficult to transfect using traditional transfection methods, and it is even harder to transfect and maintain knockdowns in Tregs. We then used siRNA for transient knockdown of SRC-3 in Tregs from six donors. Using optimized electroporation conditions, the siRNA induced knockdown decreases, albeit less effectively, the transcript levels of *SRC-3* along with *FOXP3* and *CD25* at 24 h. (Fig. 2C, data not shown). Similarly, treatment of freshly isolated human Tregs from six individuals with a known SRC-3 inhibitor, SI-2 ((1E)-1-(2-pyridinyl)-ethanone-2-(1-methyl-1H-benzimidazol-2-yl)hydrazone)^22^ showed reduced transcript levels of *FOXP3* and *CD25* in most donors (Fig. 2D). To access whether SRC-3 inhibition affected Treg markers at the protein level, we performed fix/perm antibody staining on stimulated Tregs treated with SI-2^22^ or DMSO vehicle control. In a total of five experiments from separate donors, we consistently observed significantly decreased FOXP3 and CD25 staining after SI-2 treatment using standard flow cytometry (Fig. 2E and See Supplementary Fig. 2A online, n = 3) or imaging cytometry (Fig. 2F and See Supplementary Fig. 2B online, n = 2). These data support an active role for SRC-3 in Treg gene regulation and suggest that its perturbation may influence Treg induction and function.

**Figure 3:**
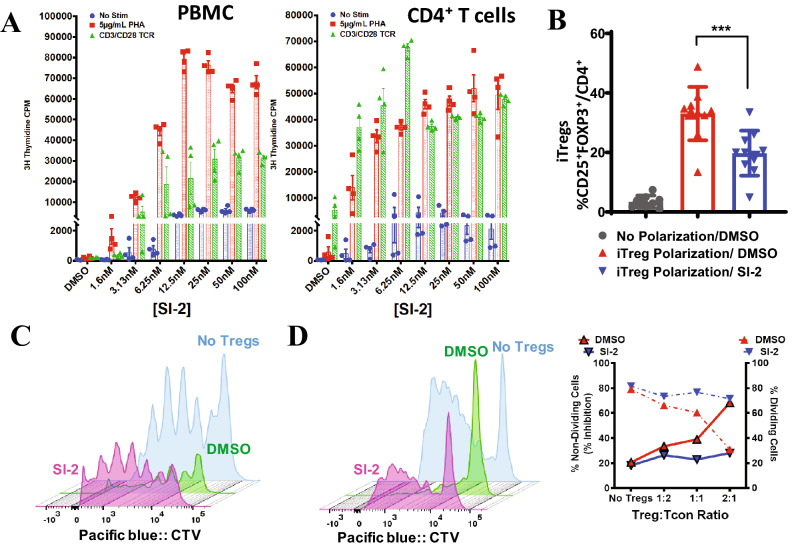
Inhibition of SRC-3 abrogates Treg suppression function. (**A**) Low-dose inhibition of SRC-3 with SI-2 promotes the proliferation of PHA and CD3/CD28 stimulated human PBMC and bulk CD4 + T cells. (**B**) Inhibition of SRC-3 activity in human resting T cells blocks induction into iTregs and (**C**) restricts their ability to suppress proliferating T cells. (**D**) SRC-3 inhibition in Tregs directly isolated from human blood donors relieves their suppressive potential.

Coste et al.^9^ previously reported that isolated T cells from SRC-3 knockout mice had increased basal and CD3/CD28-induced proliferation, a phenomenon that could be rescued by transfection of SRC-3 cDNA. In the context of our new discovery of a transcriptional role for SRC-3 in Tregs, we thus sought to test whether acute perturbation of SRC-3 would affect proliferation of human blood cell populations. Again, low dose treatment with SI-2, our established SRC-3 inhibitor, dramatically elevated the proliferation of human PBMCs, bulk T cells, and bulk CD4 cells (Fig. 3A and See Supplementary Fig. 3A online). Next, we used induction of iTregs from resting CD4 cells as an assay to probe SRC-3 function during this cell type transition. We consistently observed significantly reduced numbers of FOXP3 + /CD25 + cells when SRC-3 was inhibited during iTreg induction of resting CD4 cells (Fig. 3B) from nine healthy donors spanning a broad range of age and genders (See Supplementary Fig. 3B online). Cell viability, size, and shape are unchanged by SRC-3 perturbation, indicating that the effect is not due to toxicity (See Supplementary Fig. 3C online). Unsorted iTregs from these experiments were capable of suppressing the proliferation of CellTrace Violet (CTV) labeled CD4 cells from the same donor, but this suppression was lost if SRC-3 was inhibited during the iTreg differentiation (Fig. 3C and See Supplementary Fig. 3D online). To further determine if the proliferative effect of SRC-3 inhibition can be distilled down to isolated populations, we performed co-culture experiments using freshly isolated CD4 + CD25 + (Tregs) and conventional CD4 + CD25- (Tcon) cells. Tregs were treated for 18 h with SRC-3 inhibitor or vehicle and extensively washed to remove drug, then counted for viability using trypan blue exclusion. These unstained Tregs are mixed across a range of ratios with CTV-stained Tcon cells and CTV intensity (proliferation) was measured after five days using flow cytometry. This short-term inhibition of SRC-3 in freshly isolated peripheral human Tregs decreased their suppressive action (Fig. 3D and Suppl. Fig. 3E), indicating that acute loss of SRC-3 function in Tregs is detrimental to Treg suppressive activity. Furthermore, the washout suggests targeting SRC-3 in Tregs may be a durable strategy for blocking their activity ex vivo. Similar attempts with SRC-3 transiently knocked down Tregs also showed an effect, although minimal, on the proliferation of CTV stained Tcon cells. We reasoned that the knockdown of SRC-3 in Tregs was transient, as the suppressive effect was not observed after 5 days of proliferation in activation media (See Supplementary Fig. 3F online). More research will be needed to determine whether this nuclear strategy could have benefit in humans.

In this study, we uncovered a novel function of the steroid receptor coactivator 3 (SRC-3/NCOA3) in regulatory T cell biology—SRC-3 is required for Treg suppression activity. Mice lacking the *Src-3* gene display lymphoproliferative and hyper-inflammatory phenotypes and expanded corroboration of those studies are provided herein. Moreover, we find extensive evidence across multiple bioinformatics platforms for a significant enrichment of SRC-3 coactivator in Tregs. These findings translate to human samples, where we additionally find that SRC-3 is required for efficient Treg induction as well as maintenance and function (Fig. 3).

## Discussion

The implications of a gene evolutionarily hijacking suppressive immune cells are intriguing, but the fact that this example involves a transcriptional coactivator is especially important since most current work centers on membrane/receptor/ligand regulation. Transcription is the key regulatory point in for gene expression. However, transcription factors and transcriptional coactivators are necessary for precise and temporal gene regulation and organism homeostasis across multiple cell types. The role of SRC-3 as a driver of the function of suppressive immune cells is important, as should be the regulation of Treg biology at a transcriptional level. The behavior of the SRC-3 coactivator perhaps can be understood in the context of evolution, where a venal gene corrupts cellular programs to achieve self-replication. However, there also is selective pressure from the immune system actively seeking to destroy aberrant self-cells. To this end, SRC-3 is an oncogene which modulates cell cycle control, cellular proliferation, tissue invasion and metastasis in cancer cells while also controlling the immune system via downregulating its response. SRC-3 appears to be linked to both of these concepts, ultimately providing a broad permissive environment for (cancer) cells to proliferate while being shielded from immune surveillance. To further understand the role of SRC-3 in immune biology of cancer, we may also need to study the variable expression levels of SRC-3 in different subtypes of Tregs, especially those present in TME and versus ones circulating in the periphery.

For SRC-3, this axis also may have evolved as a contributory means for Tregs to mitigate maternal–fetal immune system conflict^23,24^. Whole-body *Src-3* knockout mice are sub-fertile, which has been generally attributed to the coactivator’s role as a sex steroid receptor coactivator in the female reproductive track^12^. Based on the current study, one could hypothesize that SRC-3 action in maternal Tregs may play a role in part to protect the fetus from the maternal immune system. Dysregulation of SRC-3 also may play a role in hyperimmune syndromes and autoimmune diseases. Perhaps most importantly, the realization of the critical role that SRC-3 plays in Treg function, suggests a potential new ‘intracellular nuclear transcriptional approach’ to immune checkpoint therapy for cancer treatment.

## Material and methods

### Mice

Five-week-old normal (C57BL/6 J) mice were purchased from Jackson Laboratory. SRC-3^-/-^ mice were generated in house and have been previously described^9,10,12^. The animal studies were carried out in accordance with the National Institutes of Health guidelines and were granted approval by BCM’s Institutional Animal Care and Use Committee. All animal studies were performed in compliance with the ARRIVE guidelines.

### Mouse splenocytes isolation and immunophenotyping

Mouse spleen single-cell suspensions were prepared by using collagenase and DNase digestion with gentleMACs tissue disruption in C-Tubes. Resulting suspension was filtered (40um mesh), RBC Lysed, and FcBlocked prior to staining. The single cell splenocytes suspensions were analyzed by using BD LSRII for antibody panel established by the Cell Cytometry and Sorting Core at Baylor College of Medicine in collaboration with the International Mouse Phenotyping Consortium. Surface antibodies included in this study are—CD5, CD4, CD44, CD8, CD25, CD161, CD62L, CD19, Ly6C, Ly6G, CD21, CD35, CD11b, CD11c, IA-E and CD23.

### Human subjects

The Institutional Review Board at Baylor College of Medicine reviewed the study and decided it does not qualify as human subject research. Human peripheral blood mononuclear cells (PBMCs) and T cells were obtained from blood buffy coats of healthy donors provided by Gulf Coast Regional Blood Center, Houston, TX.

### Isolation of CD4 + and CD25 + cells from human blood

Isolation of CD4 + cells from buffy coat obtained from healthy blood donors was performed using the RosetteSep Human CD4 + T cell enrichment kit as per the manufacturer’s suggested protocol. Positive selection of CD4 + CD25 + (Treg) cells was performed using MACS CD25 MicroBeads and non-selected cells were labelled as CD4 + CD25- (Tconv) cells. CD4 + resting T cells were negatively selected using CD30 MicroBeads by MACS. Purity and viability of isolated Tconv and Tregs cells were determined by flow cytometry.

### Flow cytometry and antibody staining

First, cells were rinsed with FACS buffer and then stained for the cell surface proteins CD4 and CD25 for 30 min at 4 °C. The cells were then fixed and permeabilized by using the fixation/permeabilization kit followed by the Foxp3 antibody incubation for one hour. Cells were subsequently washed and resuspended in FACS buffer for flow cytometry analysis. Before subjecting cells to FACS analysis, all the samples were passed through a 0.40 μm filter and stained with SYTOX Orange Dead Cell Stain (invitrogen). Samples were run on a BD FACSCanto II (Becton and Dickinson) and sorted with a FACSArial cell sorter (Becton and Dickinson) using the Diva software package. Analyses were performed with either Diva or FlowJo_v10 software. Graphs were generated with Prism 8 software (GraphPad Software, Inc.). The antibodies used for staining were anti-human/ mouse CD4 FITC, anti-human CD25 APC, and anti-human Foxp3 PE (eBioscience). Proper controls were also used through the sample preparation process, including a negative control and single-color controls for each antibody.

### Preparation of cell lysates and immunoblotting

Briefly, cells were lysed with NP-40 lysis buffer with phosphatase and protease inhibitors at 4 °C for one hour. Samples were then centrifuged at 17,000×*g* for 10 min and protein concentration was determined using the Bradford assay. Samples were loaded into NuPAGE 4–12% Bis–Tris gels for protein separation, followed by standard methanol transfer to nitrocellulose membrane at constant 30 mA overnight. The membranes were then blocked in 5% fat free milk diluted in PBS with 0.5% Tween-20 (PBST) for one hour at room temperature. Primary and secondary antibodies were diluted in 1% fat free milk in PBST and all performed at 1:1000 except for β-actin which was used at 1:5000. Primary antibody incubation times were done for either 4 h at room temperature or overnight at 4 °C. Secondary antibodies conjugated to horseradish peroxidase were used at 1:5000 and incubated for one hour at room temperature in 1% fat free milk in PBST. Membranes were then reacted with ECL or Femto reagents, allowing chemiluminescence detection using a GE Amersham imaging system.

### RNA extraction and quantitative reverse-transcription PCR

Total mRNA was isolated from cells using TRIzol to lyse the cells followed by RNA phase separation isolation method and GlycoBlue precipitation. cDNA was then reverse transcribed from the isolated RNA by using Vilo SuperScript Master Mix cDNA synthesis kit (Invitrogen). To study relative gene expression, qPCR was carried out using sequence specific primers and SYBR green chemistry. Relative mRNA expression was calculated by the ΔΔCT method with normalization to 18S rRNA. Each result is represented by at least three technical replicates.

### Transfection and transduction

For knockdown, small-interfering RNA (siRNA) was custom designed and/or ordered as pre-validated duplexes. Lipofectamine 2000 (Life technologies) was used to transiently transfect plasmid DNA and siRNA into cells. For luciferase reporter assay, the cells were harvested using Tris–EDTA-NaCL (TEN) buffer, centrifuged at 350×*g* to pellet, and lysed using passive lysis buffer. Reporter activity was analyzed using a Berthold LB 960, and RLU was normalized to protein quantity of each sample.

The electroporation knockdown was carried out using the Biorad Gene Pulser Xcell. Parameters for the Gene Pulser were optimized by varying the voltage and concentration of knockdown constructs per manufacturers recommendations. Cells were washed briefly with PBS, and resuspended at a concentration of 10 million cells per milliliter in PBS. Once the siRNA or DNA was added to the cells, the cells were immediately electroporated. After electroporation, warm culture media was added directly to the cells to bring the concentration to 5 million cells per mL and incubated at 37 °C for ten minutes before downfield applications.

Lentiviral vector was produced by transient transfection of 293 T cells using Lipofectamine 2000 and packaging plasmids pMDG and ΔNRF. After 48 h, supernatants with the lentivirus were harvested for purification and precipitation using PEG-it Virus Concentration Solution. Spinfection protocol was used to transduce primary T cells. In summary, the primary cells were suspended in complete media with polybrene (8ug/mL), pyruvate (100 nM), and nucleosides adenosine, guanosine, cytosine and thymidine (50uM) and centrifuged for 90 min at 2000 × g at room temperature. Cells were resuspended in complete T cell media with IL-2 (50U/mL) after the centrifugation.

### Induction of Tregs (iTregs) from resting CD4 T cells

Induced Tregs (iTregs) were generated from resting CD4 + CD30- by incubating in a cocktail mixture of ImunoCult Human CD3/CD28 T cell activator, αIL-4 (250 ng/mL), αIL-12 (250 ng/mL), αIFNγ (250 ng/mL), TGFβ (2.5 ng/mL) and IL-2 (50 units/mL) for 72 h. Induction of the iTregs was confirmed on the third day through surface marker CD25 and intracellular FOXP3 staining using flow cytometry analysis.

### CTV staining and Treg co-culture

Cell trace violet (CTV) staining was carried out per company standard protocol the night before cells were co-cultured. CTV was added at a concentration of 5 mM for the staining procedure per 1 million. For the co-culture set up, cells were stimulated with either PHA (5ug/mL) or with Immunocult Human CD3/CD28 T cell activator and plated in a 96 well U- bottom plate.

### Tritiated thymidine incorporation

3H-labeled thymidine (Perkin Elmer) was added to the media of suspension cells at a density of 1 × 105 per well and then the cells were grown for 12 h. The cells were recovered using a cell harvester that quantitatively transfers to a glass fiber filtermat from 96 well plates. The filters were then dried before being transferred into plastic holders, and filled with scintillation fluid according to manufacturer’s directions. Using a beta scintillation counter, the filters were then read and counts per minute were measured for analysis.

### Luciferase assay

Briefly, HEK-293 cells were seeded into 12-well plates at a density of 5 × 10^4^ per well the day before transfection. Transfection master mixes were prepared for triplicate wells each receiving 500 ng pGL-Foxp3-P^25^ and a 375 ng sum of balancing pSG5 empty vector with 125, 250, or 375 ng of pSG5-SRC-3 plasmid expressing the human SRC-3 coding sequence. Approximately 24 h after transfection, cells were harvested in 1X Passive Lysis Buffer (Promega) and quantified using Promega Luciferase Assay System (cat. E1500). Measurements were read on a Berthold LB 960 luminometer.

## Statistical analysis

All statistical analysis results represent the mean ± SD, unless indicated otherwise. Statistical comparisons were performed using the two-tailed Student’s *t*-test and one-way or two-way ANOVA with suitable corrections for multiple comparisons. In all cases, a *p*-value of < 0.05 was considered statistically substantial and results reported were calculated from at least three independent experiments. NS, non-specific. *p < 0.05, **p < 0.005, ***p < 0.0005 by Student’s t-test. Data analysis was performed using GraphPad Prism software version 8.4.2 (GraphPad Software).

## Supplementary Information

Supplementary Information 1.

Supplementary Information 2.
