# Supplementary material for: Steroid receptor coactivator 3 (SRC-3/AIB1) is enriched and functional in mouse and human Tregs
Source: Sci Rep. 2021 Feb 9;11:3441. doi: 10.1038/s41598-021-82945-3 (PMC7873281; doi:10.1038/s41598-021-82945-3)

Supplementary material for

Title: Steroid Receptor Coactivator 3 (*SRC-3/AIB1*) is enriched and functional in mouse and human Tregs

Authors and affiliations: Bryan C. Nikolai[1,4*]†, Prashi Jain[1,4]†, David L. Cardenas[1,4], Brian York[1], Qin Feng[1,2], Neil J. McKenna[1,4], Subhamoy Dasgupta[1,3], David M. Lonard[1,4], and Bert W. O'Malley[1,4*]

[1] Department of Molecular and Cellular Biology, Baylor College of Medicine, Houston, TX 77030

[2] Center for Nuclear Receptors and Cell Signaling, University of Houston, Houston, TX 77204

[3] Department of Oncology, Roswell Park Comprehensive Cancer Center, Buffalo, NY 14263

[4] Laboratory of Molecular Regulation, Baylor College of Medicine, Houston, TX 77030

† These authors contributed equally

Running Title: SRC-3 enrichment and function in Tregs

This file includes three supplementary figures and 4 full western blot images

A.

CD5
CD4
CD44
CD8
CD25
CD161
CD62L
CD19
Ly6C
Ly6G
CD21
CD35
CD11b
CD11c
IA-E
CD23

B.

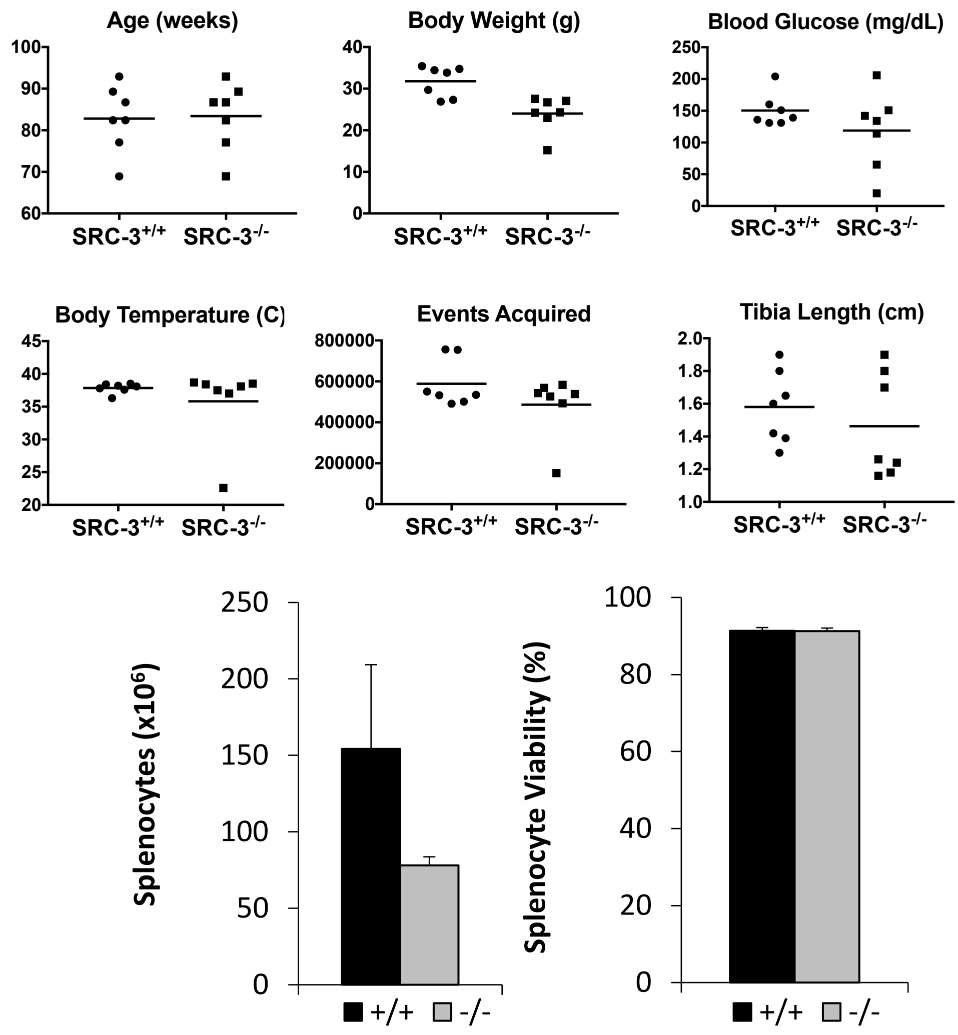

Fig. S1: A) Antibody panel used in the immune phenotyping studies and established by the Cell Cytometry and Sorting Core at Baylor College of Medicine in collaboration with the International Mouse Phenotyping Consortium. B) Metadata for age, body weight, blood glucose level, body temperature, number of FACS events, tibia length, splenocytes number and viability of spleens from src-3 knockout mice and age-matched littermates (n=7).

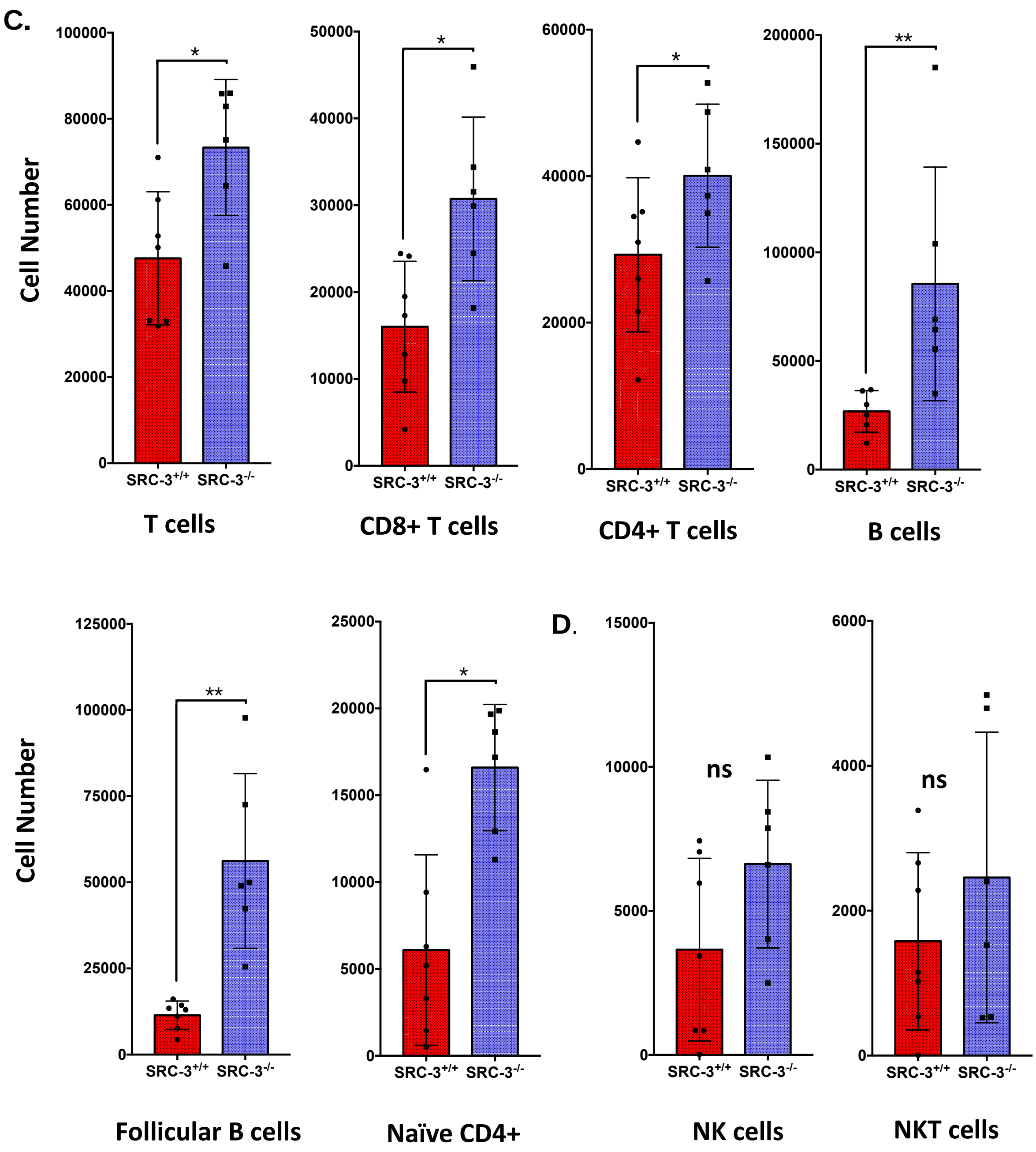
C.
Cell Number
*
**
SRC-3+/+
SRC-3-/-
T cells
CD8+ T cells
CD4+ T cells
B cells
Follicular B cells
Naïve CD4+
D.
ns
NK cells
NKT cells

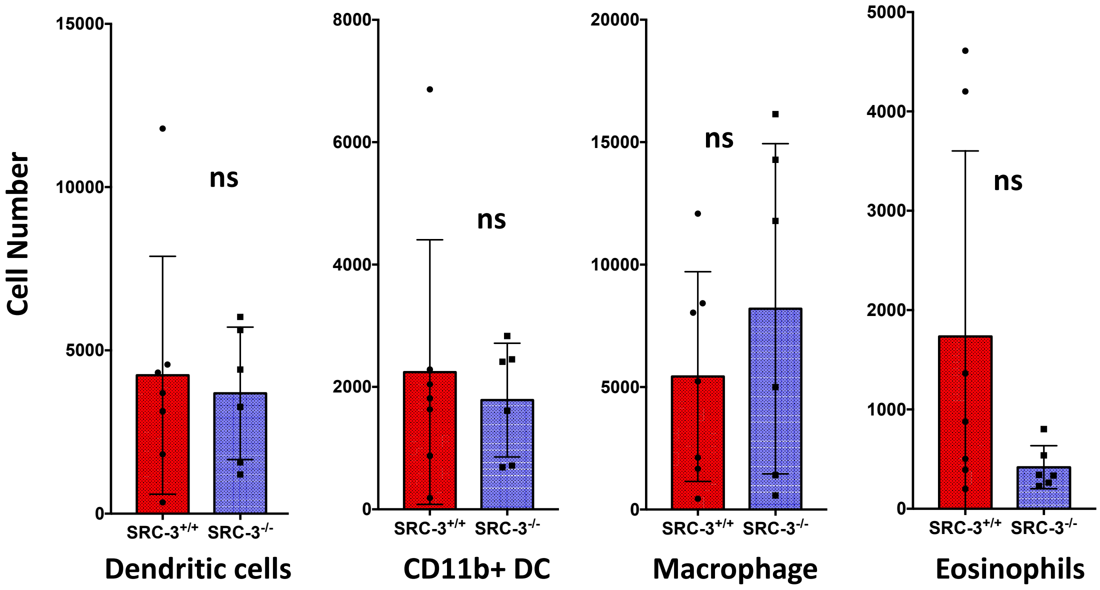

Fig. S1: C) SRC-3 is enriched in T cell resident organs and SRC-3 KO mice have shown broad lymphoproliferation in T-cells, CD8+ T-cells, CD4+ T-cells, B-cells, Follicular B cells, as well as in Naïve CD4+ T-cells. D) Natural killer (NK) cells, NKT, dendritic, macrophages and eosinophils cells are remains largely unchanged or slightly decreased in SRC-3 KO mice compared to the WT mice.

E.

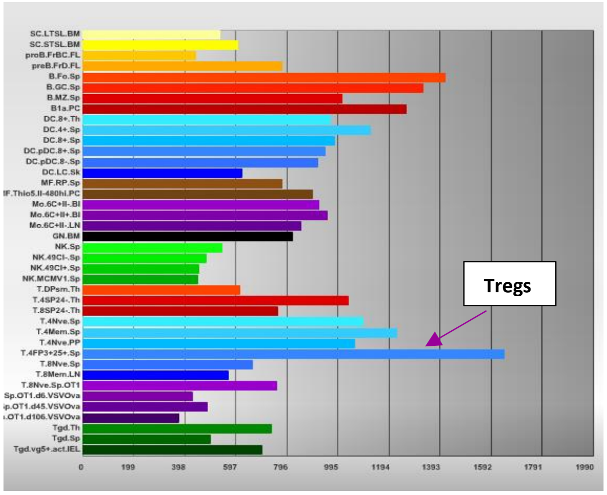

F.

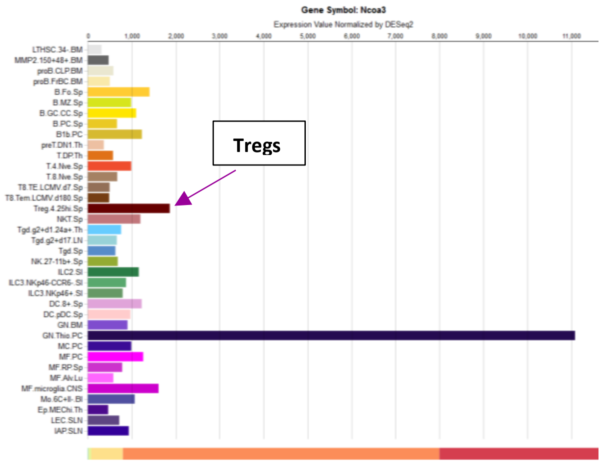

Fig. S1: **E)** Microarray as well as recent RNA sequencing data from Immune Genome project (Imm Gen) suggests that SRC-3 transcript is highly enriched in regulatory T-cells (Tregs). The immune Genome project is a collaborative project to populate the gene-expression database for various immune cells. (www.ImmGen.org Data accessed on 6/12/19) **F)** Mouse BioGPS data**:** gene expression from a diverse array of normal tissues, organs, and cell lines in mice suggests that NOCA3 is present in Tregs

2A)

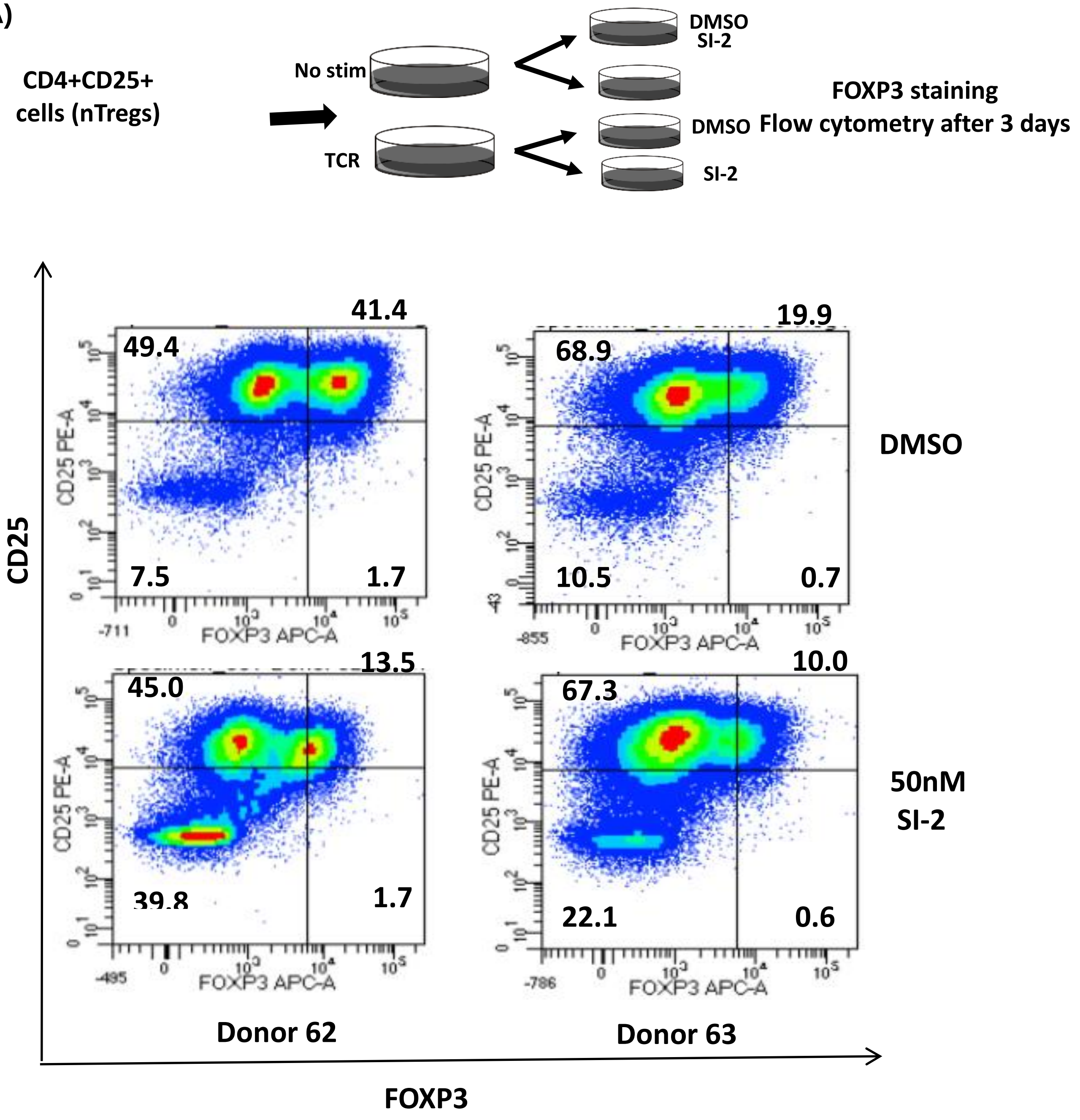

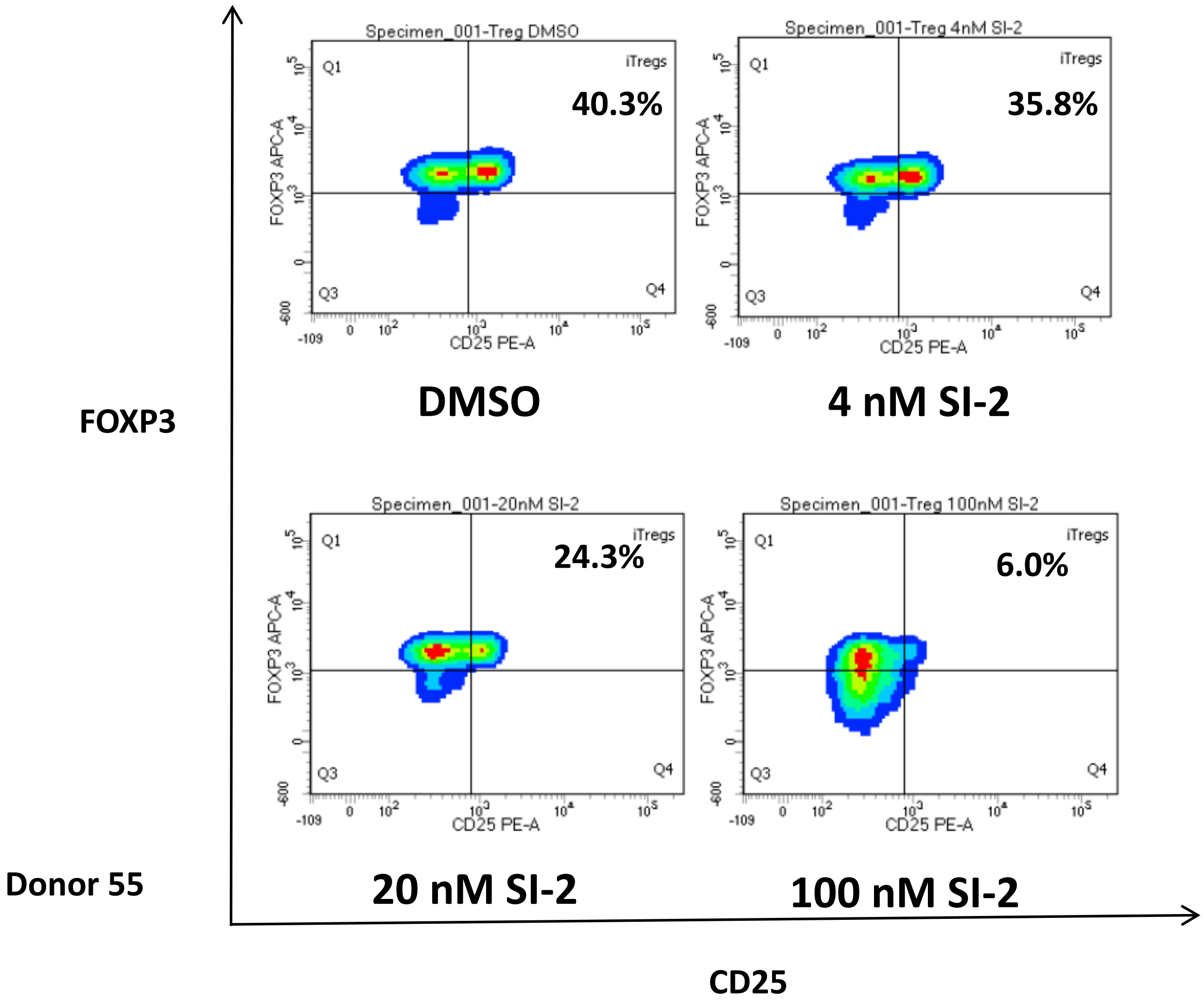

Fig. S2: Decreased Treg markers CD25 and FOXP3 in activated nTregs by SI-2, a SRC-3 small molecule inhibitor**. A)** nTregs were isolated from whole blood and treated overnight with DMSO or SI-2. The following day, cells were plated in round-bottom flasks with or without CD3/CD28 TCR stimulation and 100 IU/mL IL-2 in the presence of DMSO or SI-2. Cells were harvested after 3 days and fixed with FOXP3 and CD25 antibodies for flow cytometry. All the plots are all gated first on CD4+ cells. SI-2 blocks expression of TCR-induced CD25 and FOXP3 expression in nTregs (n=3, Donor 55, 62 and 63).

2B)

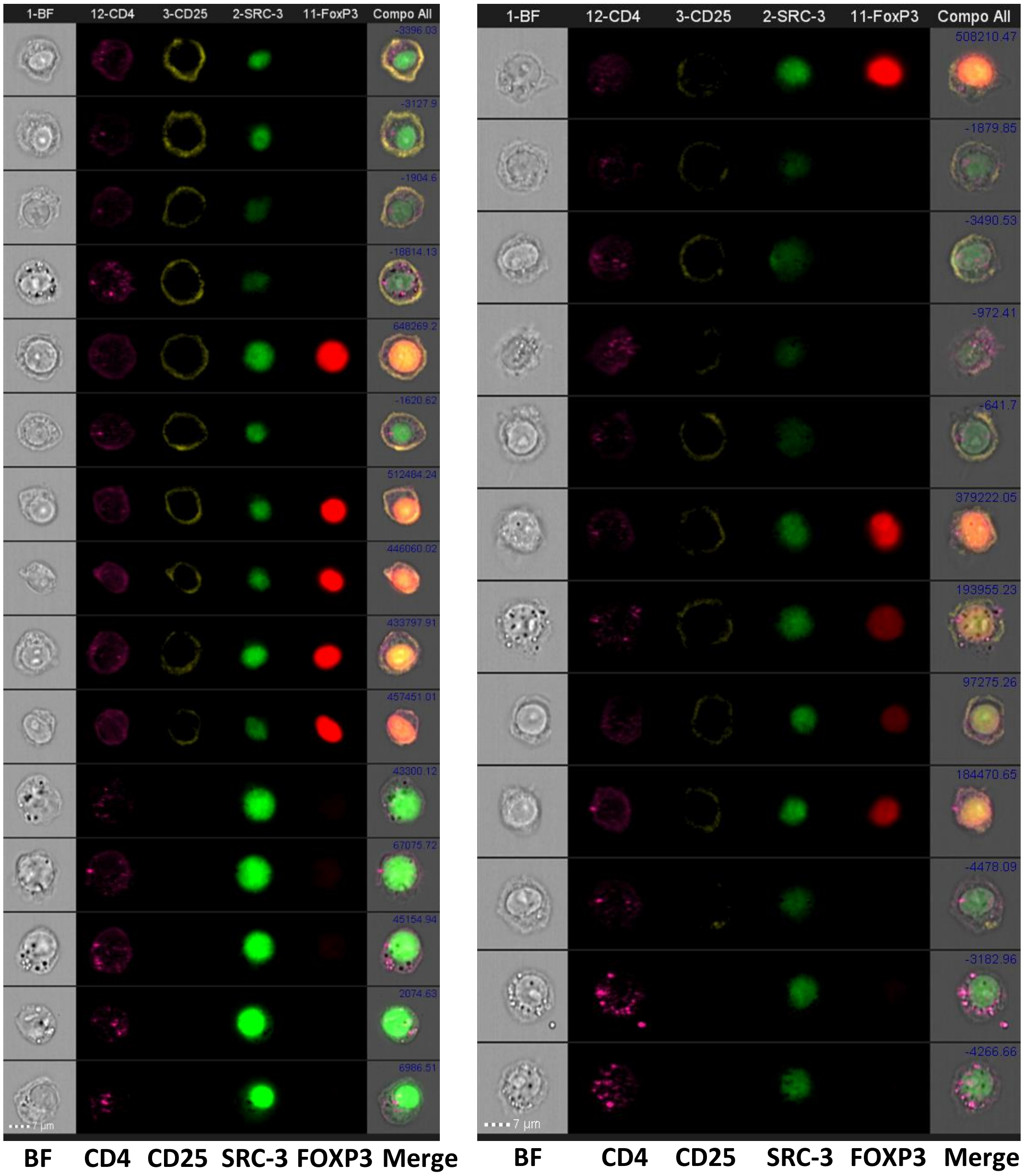

	CD25	SRC-3	FOXP3
Donor 62 DMSO	1231.6728	81021.6454	167747.8918
Donor 62 SI-2	795.9566	37896.309	70138.0393
Donor 63 DMSO	978.1983	86094.2835	114973.8368
Donor 63 SI-2	405.8439	54777.4983	52861.834

Fig. S2: Decreased Treg markers CD25 and FOXP3 in activated nTregs by SI-2, a SRC-3 small molecule inhibitor. **B)** Imaging cytometry to visualize SI-2 effect on FOXP3, CD25 and SRC-3 levels in activated Tregs (n=2, Donor 62 and 63). nTregs were isolated from whole blood and treated 2 days with CD3/CD28 TCR stimulation and 100 IU/mL IL-2 in the presence of DMSO or SI-2. Cells were harvested for flow and stained for CD4, CD25, SRC-3, and FOXP3. Samples were analyzed on Amnis ImageStream and quantified Mean Fluorescence Intensity (MFI) values are arranged in the table 1. These data conclude that SRC-3 inhibition may influence the Tregs induction and function.

3A)

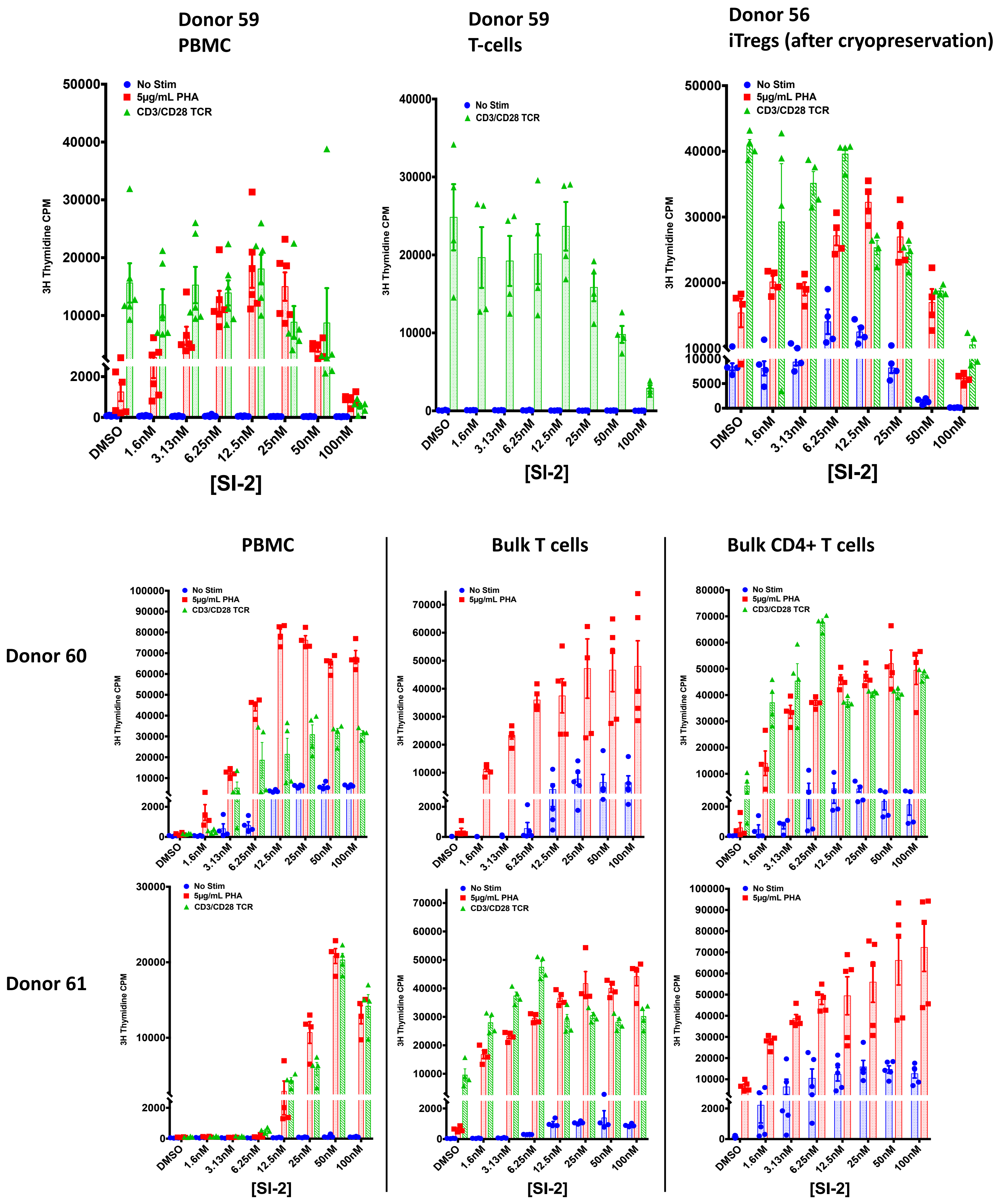
Donor 59
PBMC
Donor 59
T-cells
Donor 56
iTregs (after cryopreservation)
No Stim
5µg/mL PHA
CD3/CD28 TCR
3H Thymidine CPM
DMSO
1.6nM
3.13nM
6.25nM
12.5nM
25nM
50nM
100nM
[SI-2]
PBMC
Bulk T cells
Bulk CD4+ T cells
Donor 60
Donor 61

Fig. S3: Low dose SI-2 augments T cell mitogen-induced proliferation of human PBMCs and bulk T- cells. A) PBMCs and Total T cells were isolated from whole human blood, plated on 96-well culture plates, and rested overnight. The next day cells were treated with either no stimulator or 5ug/mL PHA (phytohaemagglutinin) or CD3/CD28 TCR stimulation. After 3rd day, tritiated (3H)thymidine was added to the culture medium for 10 hours before transfer to filter paper and scintillation counting. Data points are individual wells and error bars are SEM. (n = 4, Donors 56, 59, 60 and 61)

3B) Polarization of resting (CD4+CD30- lineage) T-cells to induced Tregs (iTregs)

Resting CD4+CD30- cells (Bulk CD4 populations) → **CD3/CD28 TCR, αIL-4 (250µg/mL), αIL-12 (250µg/mL), αIFNγ (250µg/mL), TGFβ (2.5µg/mL), IL-2 (50 IU/mL)** → **5 days** → **Induced regulatory T cells (iTregs)**

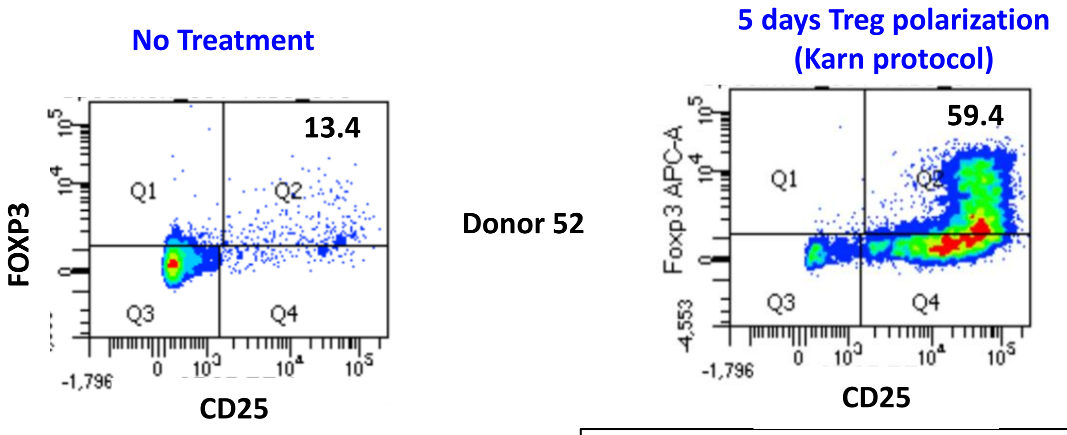

Gated first on CD4 channel

Yield: ~42 million live cells
~25 million iTregs

Method: Resting CD4+CD30- cells were isolated from whole blood and rested for overnight in IL-2 media. The following day, iTreg polarization cytokine treatment was added and 5 days later, cells were harvested. The harvested cells stained with FOXP3 and CD25 antibodies and subjected to by flow cytometry analysis. Induced Tregs showed increased FOXP3 and CD25 levels, two of the biomarkers for Tregs function.

Resting CD4+CD30- cells (Bulk CD4 populations) → **24h DMSO or 500nM SI-2** → **Plate cells** → **DMSO/SI-2 diluted to 250nM** → **Induced regulatory T cells (iTregs)**

CD3/CD28 TCR
αIL-4 (250μg/mL)
αIL-12 (250μg/mL)
αIFNγ (250μg/mL)
TGFβ (2.5μg/mL)
IL-2 (50 IU/mL)

3 days

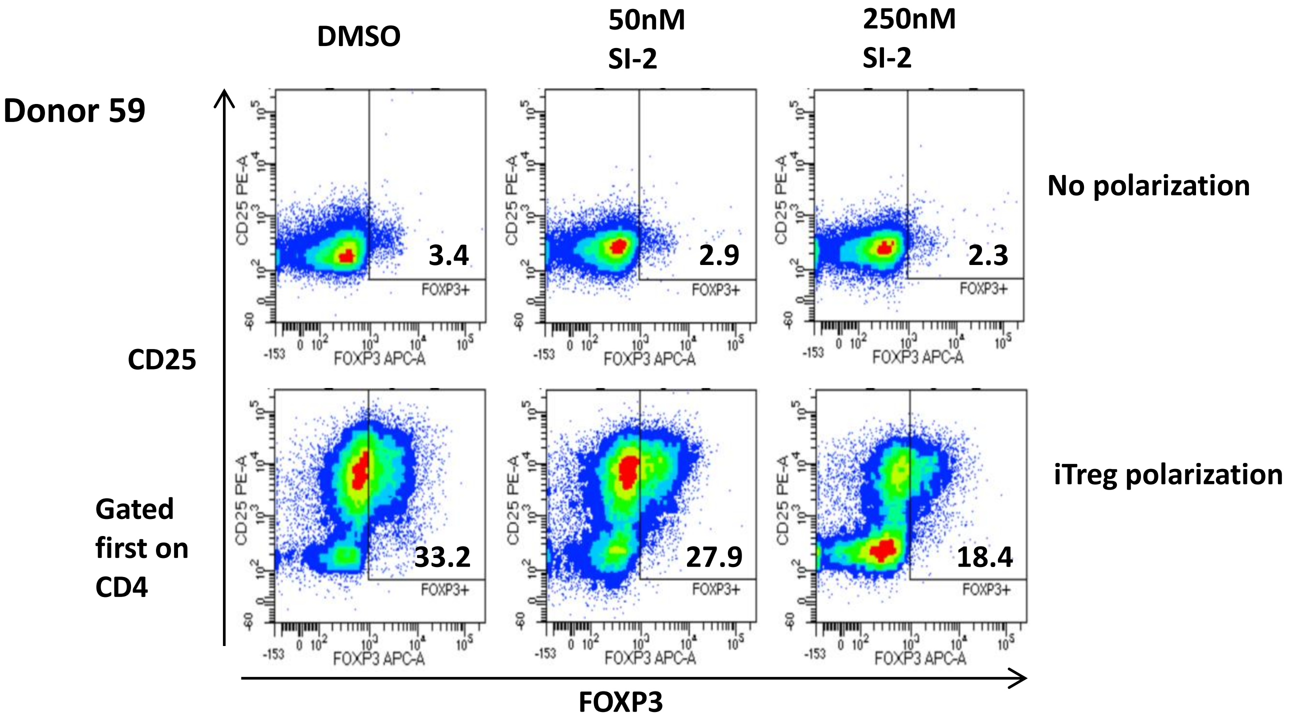

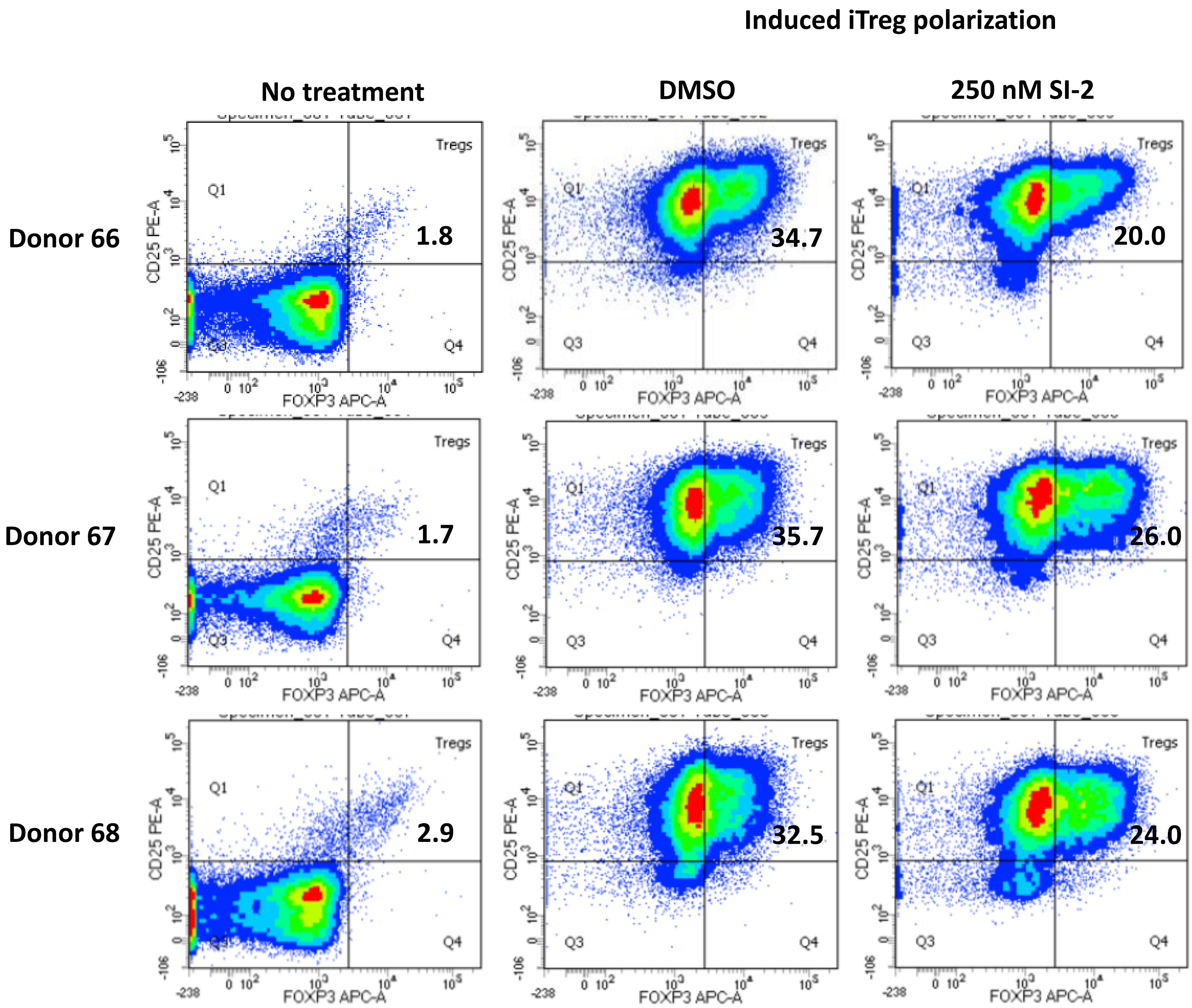

Fig S3: Proliferation assay of Tregs using various methods. **B)** Resting CD4+CD30- cells were isolated from whole blood and treated overnight with DMSO or SRC-3 inhibitor. The following day, iTreg polarization cytokine treatment was added, diluting the drug 2-fold. 3 days later, cells were harvested for flow. Induced iTreg assay from 4 human donors with considerable age and sex variation (17-60s, 1 male and 3 female). n=4 from Donors 59, 66, 67, and 68.

3C.

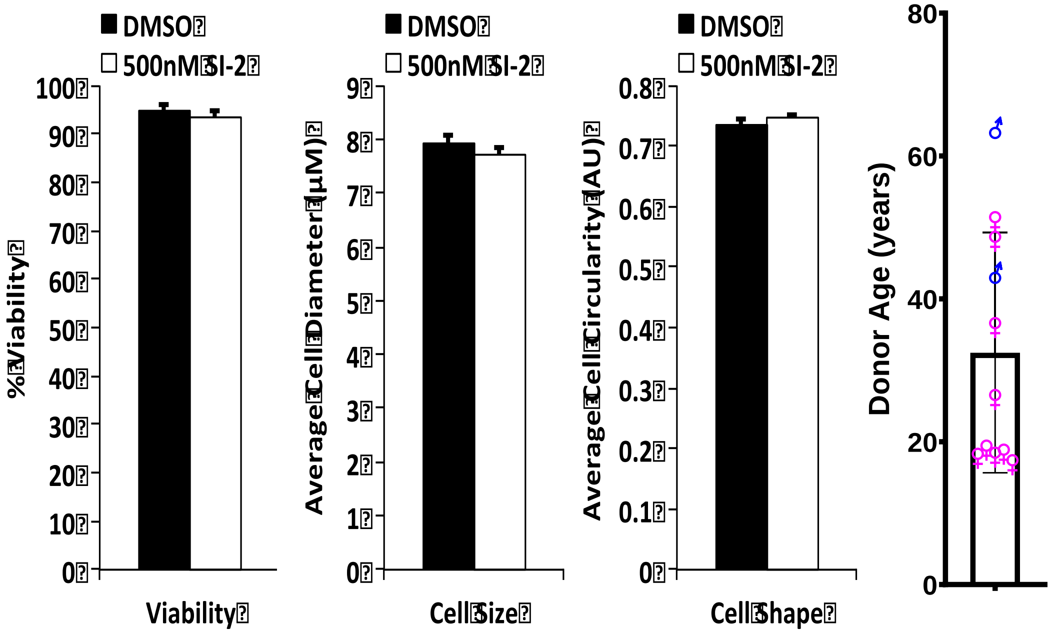

Fig S3: Proliferation assay of Tregs using various methods. **C)** The SRC-3 inhibition using SI-2 after 24 hour treatment has no effect on resting CD4+ T cell viability, size, or shape. (n=3, Donors 78, 79, and 80). Induced iTreg assay from 10 human donors with considerable age and sex variation (17-60s, 1 male and 3 female). n=10 from Donors 59, 66, 67, 68, 78, 79, 80, 87, 88 and 90.

3D). Co-culture proliferation assay using iTregs

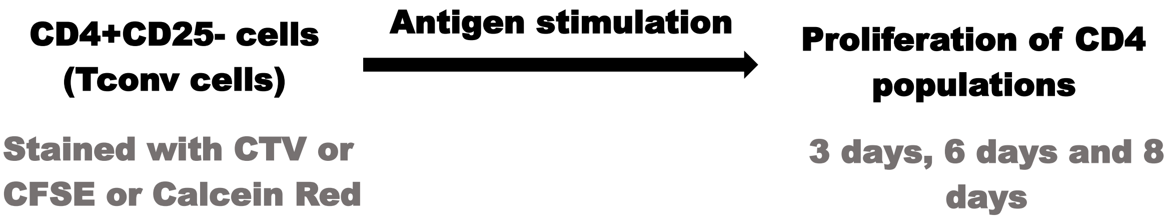

Optimization of the cell-proliferation assay with Cell Trace Violet (CTV), CFSE and Calcein orange red dye: Cells were isolated from the healthy donor as CD4+CD25- cells (Tconv cells) as well as CD4+CD30- cells(resting T cells) using the recommended manufacture's protocol. The resting T cells were polarized using the previously optimized cytokine cocktail to iTregs (Induced Tregs) over 3 days. After 3 days, cells were harvested and kept in IL-2 rich media overnight. The CD4+CD25- cells were divided into 3 pools: each pool is stained with CTV, CFSE and Calcein orange dye respectively overnight. Tconv cells were washed with culture media and plated on 96 well plate and stimulated using PHA (Phytohaemagglutinin), TCR (CD3 and CD28 antigen) and dynabeads (Dynabeads Human T-Activator CD3/CD28). The stimulated Tcells were subjected to flow cytometry for proliferation index calculations at 3, 6 and 8 days intervals. Cell Trace Violet (CTV) provided the best results after 6 days staining and stimulation by PHA.

These unsorted iTregs were tested for their suppressive nature by using proliferation of CellTrace Violet (CTV) labeled CD4 cells from the same donor. iTregs were treated for 18h with SI-2 (SRC-3 inhibitor) or DMSO and extensively washed to remove drug, then counted for viability using trypan blue exclusion. These unstained iTregs are mixed across a range of ratios with CTV-stained Tcon cells and CTV intensity (proliferation) when measured after five days. This short-term inhibition of SRC-3 in induced human Tregs decreased their suppressive action in more than 5 donors.

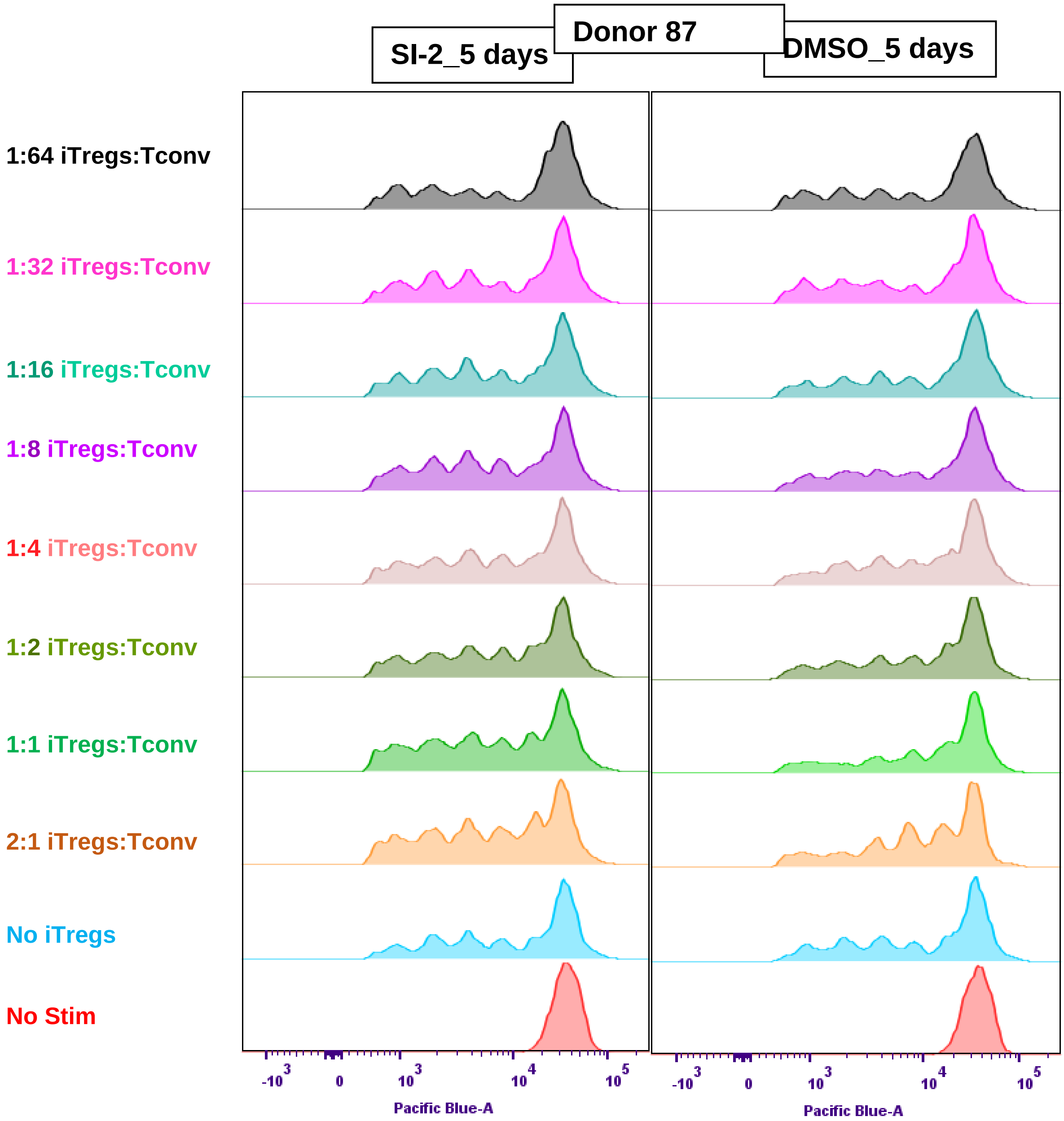
Donor 87
SI-2_5 days
DMSO_5 days
1:64 iTregs:Tconv
1:32 iTregs:Tconv
1:16 iTregs:Tconv
1:8 iTregs:Tconv
1:4 iTregs:Tconv
1:2 iTregs:Tconv
1:1 iTregs:Tconv
2:1 iTregs:Tconv
No iTregs
No Stim
-10^3
0
10^3
10^4
10^5
Pacific Blue-A
-10^3
0
10^3
10^4
10^5
Pacific Blue-A

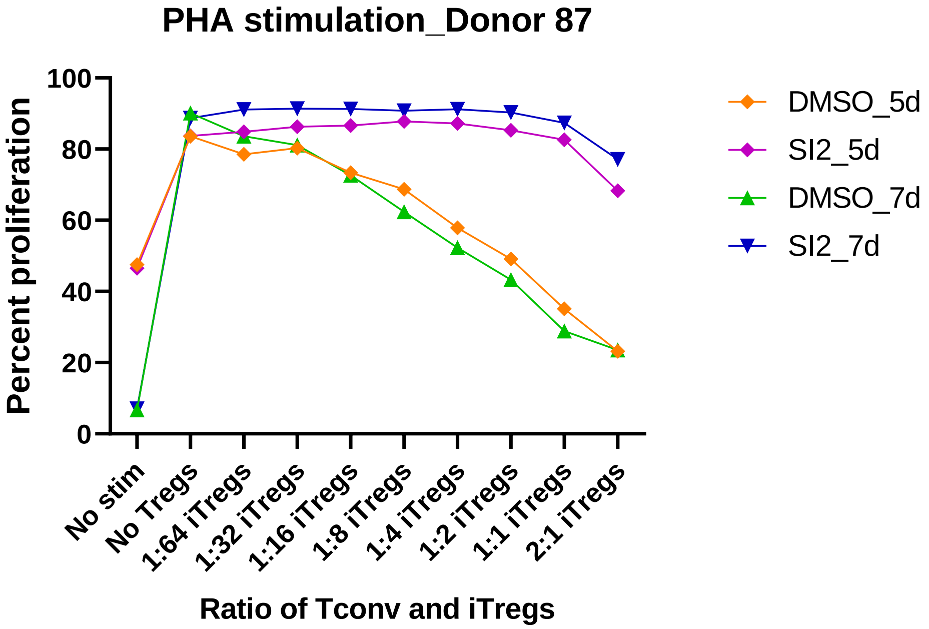
PHA stimulation_Donor 87
Percent proliferation
100
80
60
40
20
0
No stim
No Tregs
1:64 iTregs
1:32 iTregs
1:16 iTregs
1:8 iTregs
1:4 iTregs
1:2 iTregs
1:1 iTregs
2:1 iTregs
Ratio of Tconv and iTregs
DMSO_5d
SI2_5d
DMSO_7d
SI2_7d

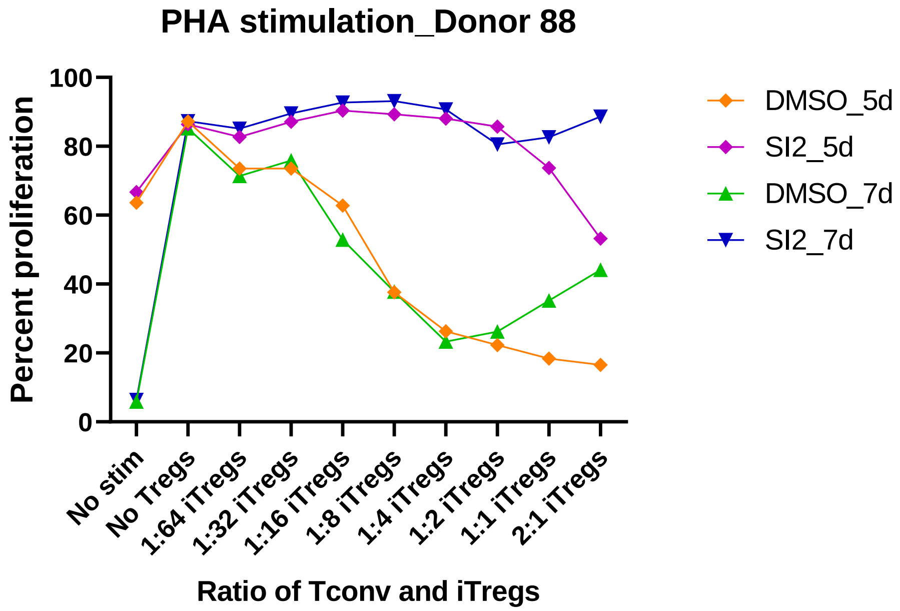
PHA stimulation_Donor 88
Percent proliferation
100
80
60
40
20
0
No stim
No Tregs
1:64 iTregs
1:32 iTregs
1:16 iTregs
1:8 iTregs
1:4 iTregs
1:2 iTregs
1:1 iTregs
2:1 iTregs
Ratio of Tconv and iTregs
DMSO_5d
SI2_5d
DMSO_7d
SI2_7d

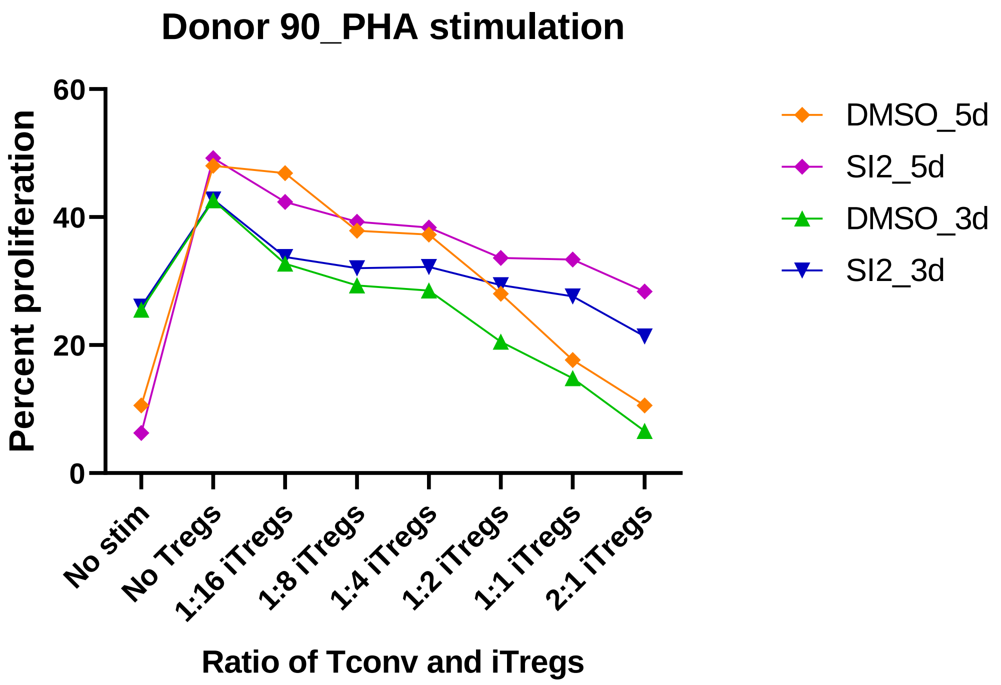

3E. Schematic for proliferation assay

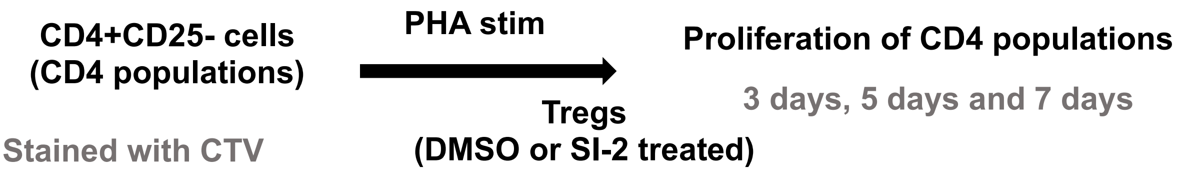

The freshly isolated peripheral human Tregs were treated with DMSO or SRC-3 inhibitor (SI-2) to see the effect of the temporary inhibition of SRC-3 on their suppressive nature. We performed co-culture experiments using freshly isolated CD4+CD25+ (Tregs) and conventional CD4+CD25- (Tcon) cells. This short-term inhibition of SRC-3 in freshly isolated peripheral human Tregs decreased their suppressive action (Supp. Fig. 3E), indicating that acute loss of SRC-3 function in Tregs is detrimental to Treg activity. We have seen similar phenomenon in 6 different healthy donors.

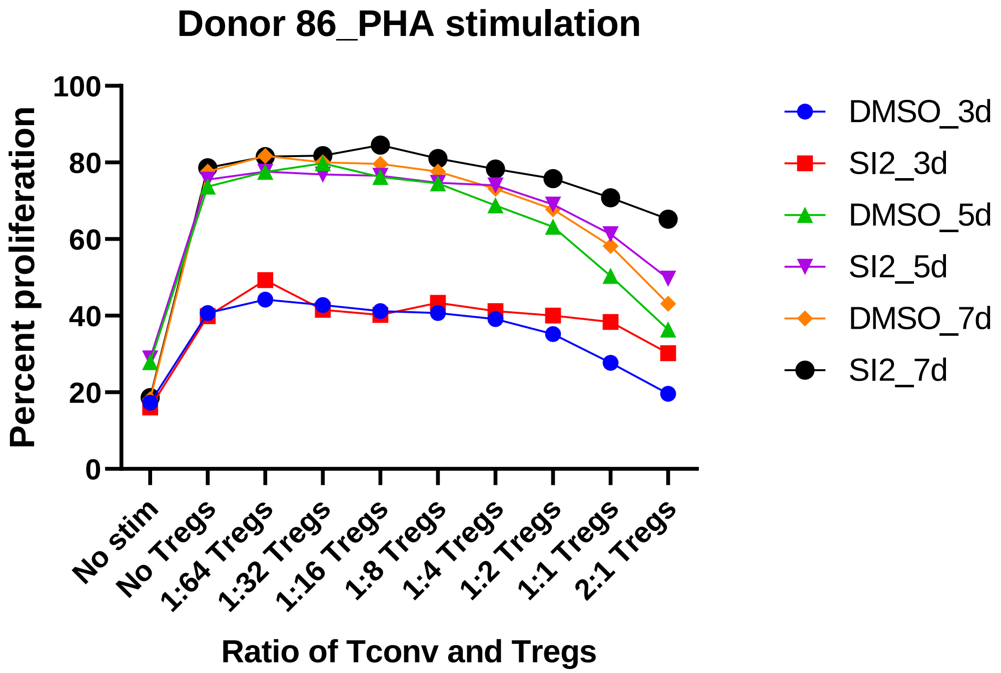
Donor 86_PHA stimulation
Percent proliferation
0
20
40
60
80
100
No stim
No Tregs
1:64 Tregs
1:32 Tregs
1:16 Tregs
1:8 Tregs
1:4 Tregs
1:2 Tregs
1:1 Tregs
2:1 Tregs
Ratio of Tconv and Tregs
DMSO_3d
SI2_3d
DMSO_5d
SI2_5d
DMSO_7d
SI2_7d

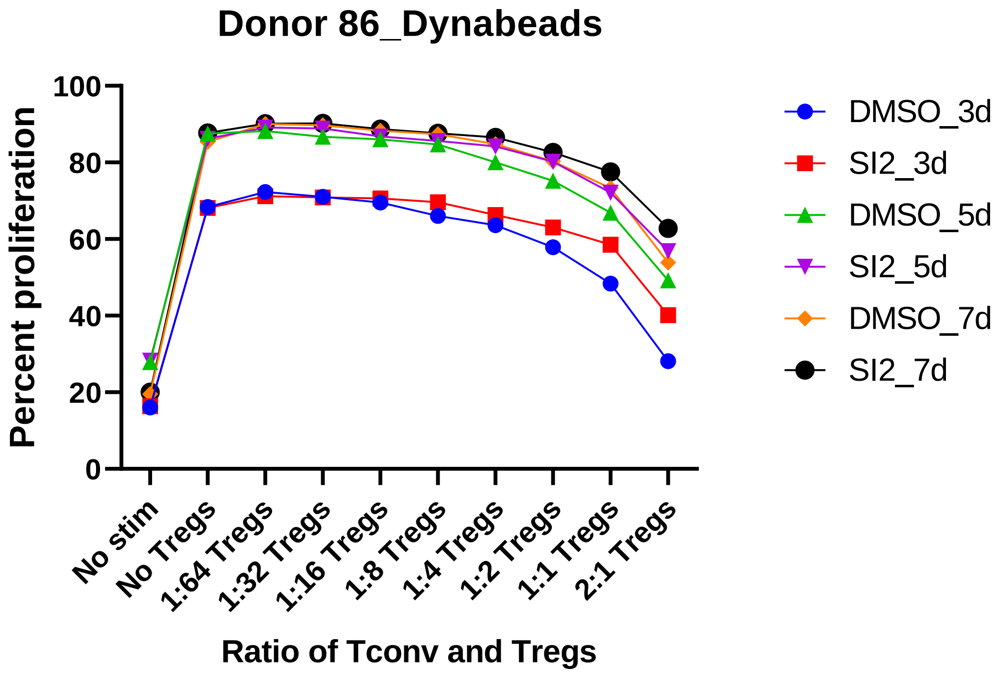
Donor 86_Dynabeads
Percent proliferation
0
20
40
60
80
100
No stim
No Tregs
1:64 Tregs
1:32 Tregs
1:16 Tregs
1:8 Tregs
1:4 Tregs
1:2 Tregs
1:1 Tregs
2:1 Tregs
Ratio of Tconv and Tregs
DMSO_3d
SI2_3d
DMSO_5d
SI2_5d
DMSO_7d
SI2_7d

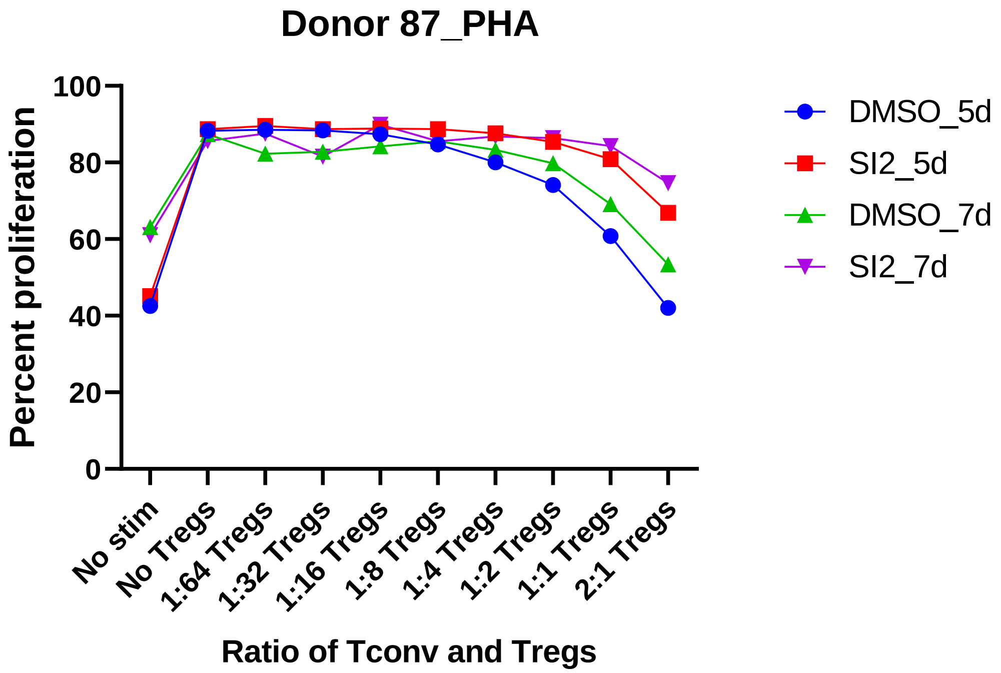

Donor 87_PHA
Percent proliferation
100
80
60
40
20
0
No stim
No Tregs
1:64 Tregs
1:32 Tregs
1:16 Tregs
1:8 Tregs
1:4 Tregs
1:2 Tregs
1:1 Tregs
2:1 Tregs
Ratio of Tconv and Tregs
DMSO_5d
SI2_5d
DMSO_7d
SI2_7d

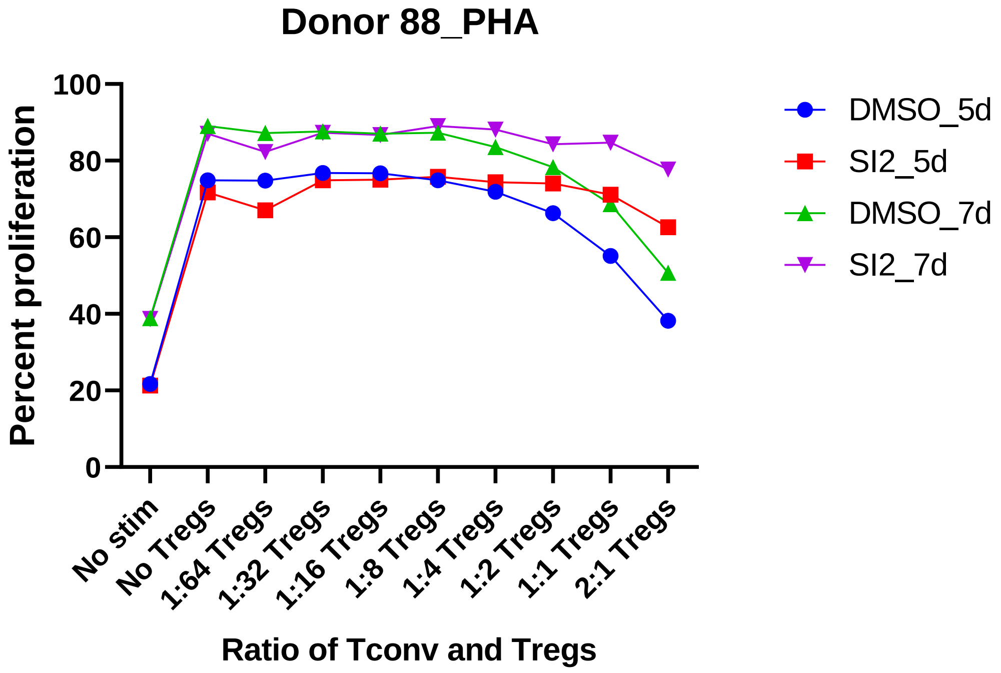

Donor 88_PHA
Percent proliferation
100
80
60
40
20
0
No stim
No Tregs
1:64 Tregs
1:32 Tregs
1:16 Tregs
1:8 Tregs
1:4 Tregs
1:2 Tregs
1:1 Tregs
2:1 Tregs
Ratio of Tconv and Tregs
DMSO_5d
SI2_5d
DMSO_7d
SI2_7d

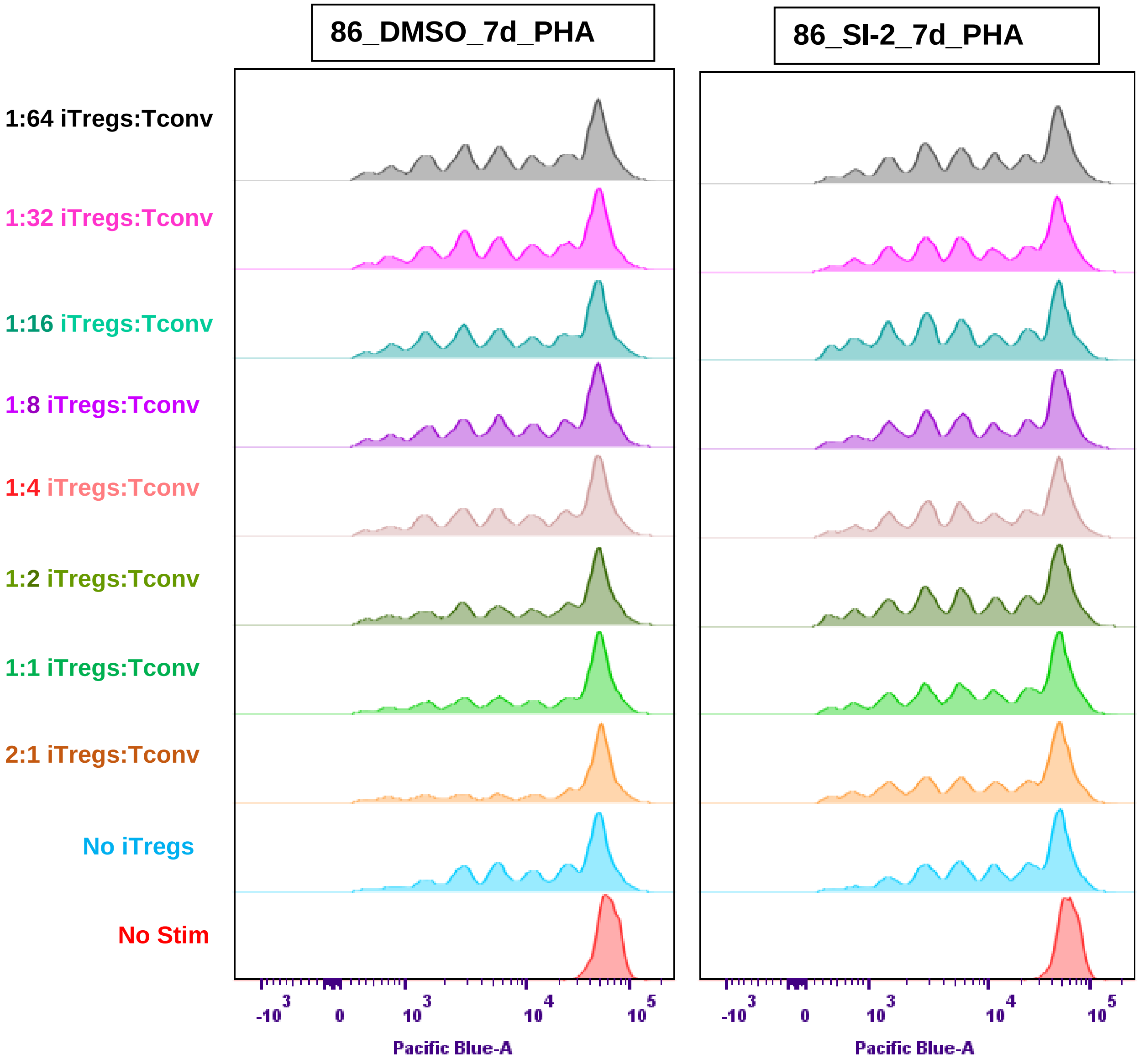

Fig S3: Pharmacological inhibition of SRC-3 disrupts suppression activity of human D) iTregs (Induced Tregs) and E) Tregs (freshly isolated Tregs)

3F)

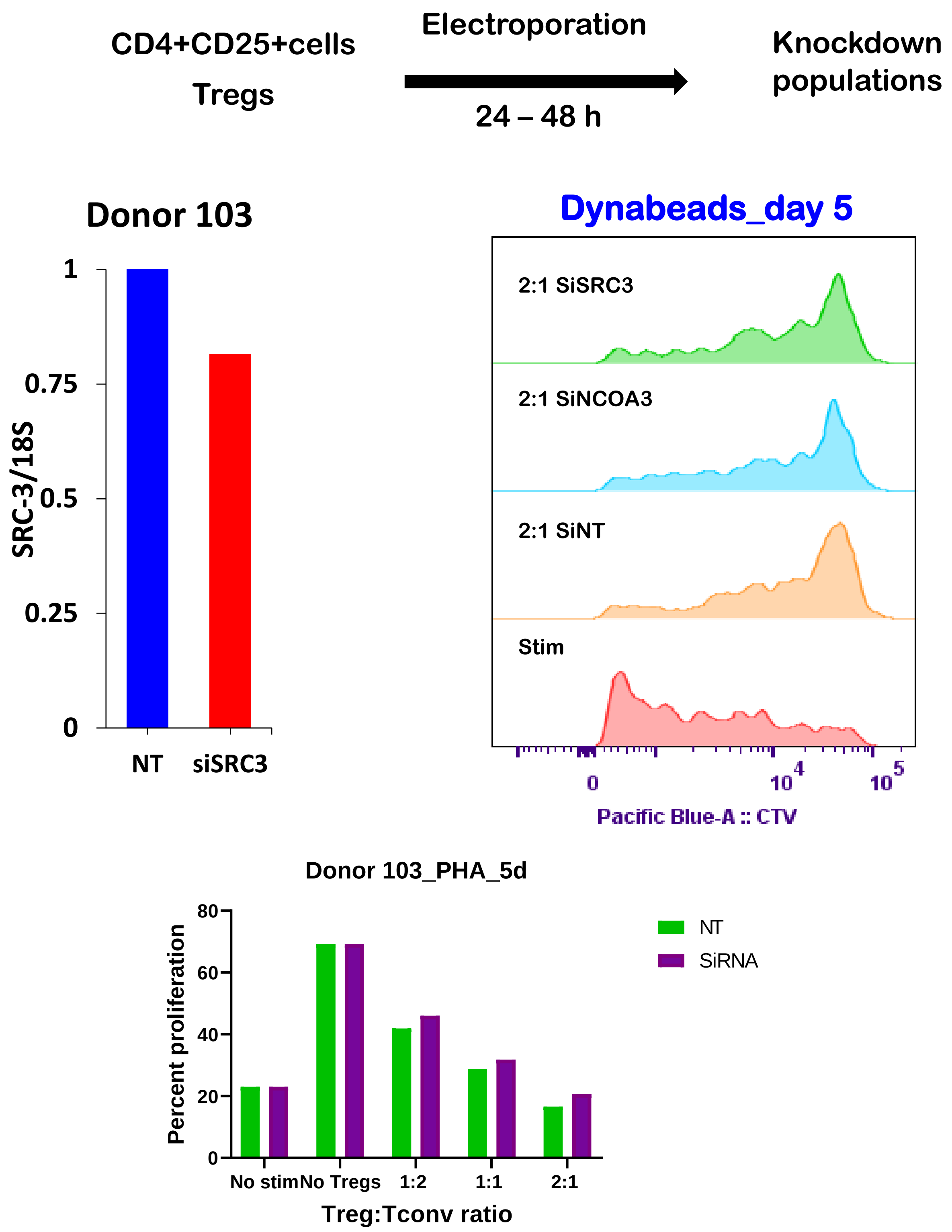

Fig S3F: Depletion of SRC-3 disrupts suppression activity of human Tregs (freshly isolated Tregs)

Main Figure 1A –Full gel image probed for SRC-3

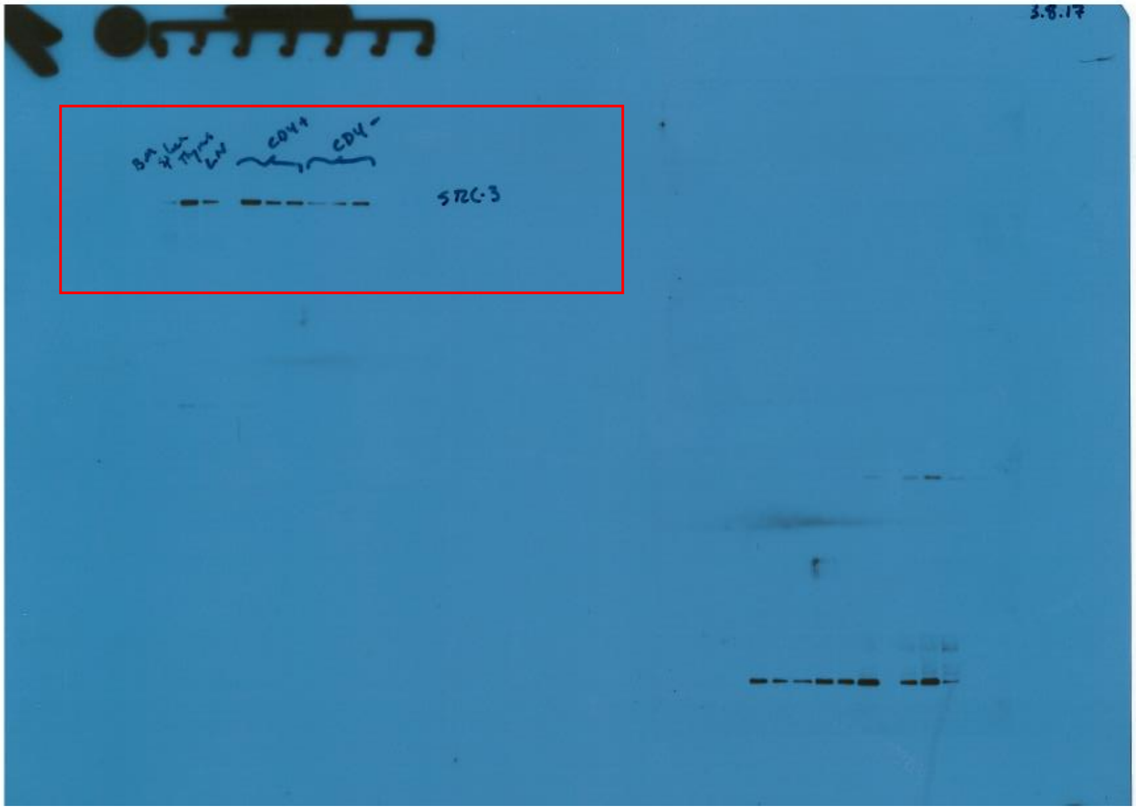

Main Figure 1A – Striped and reprobed for β-actin: Full gel image

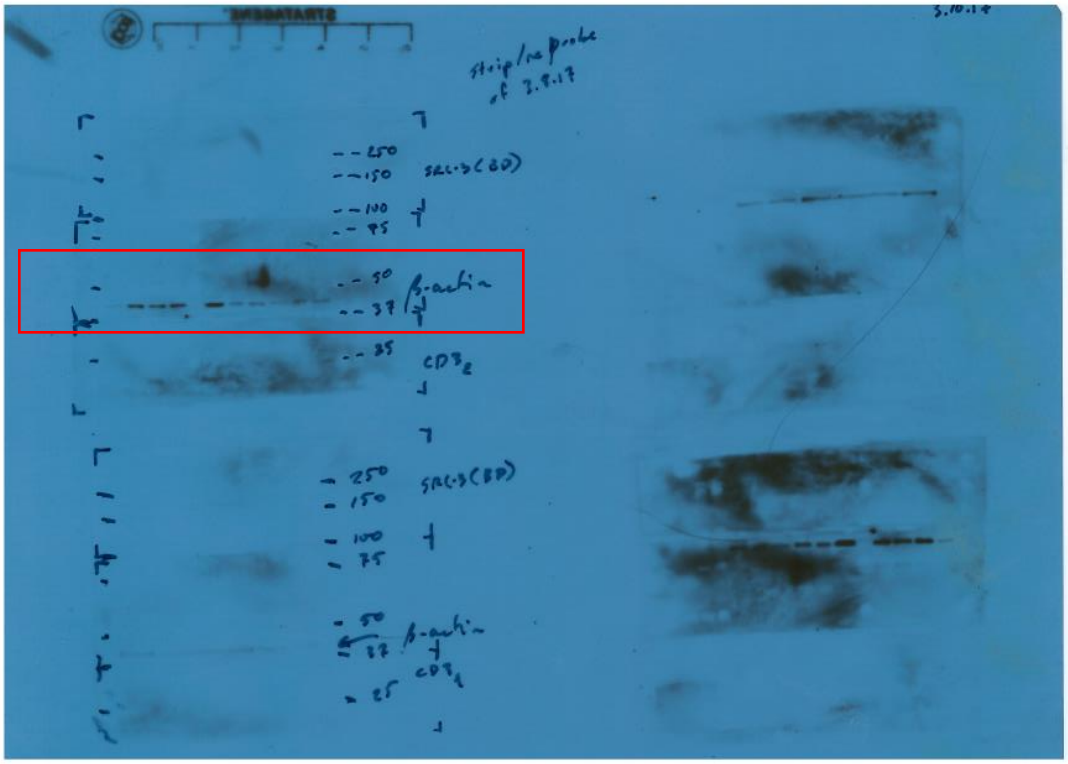

Main Figure 1D – Full gel image

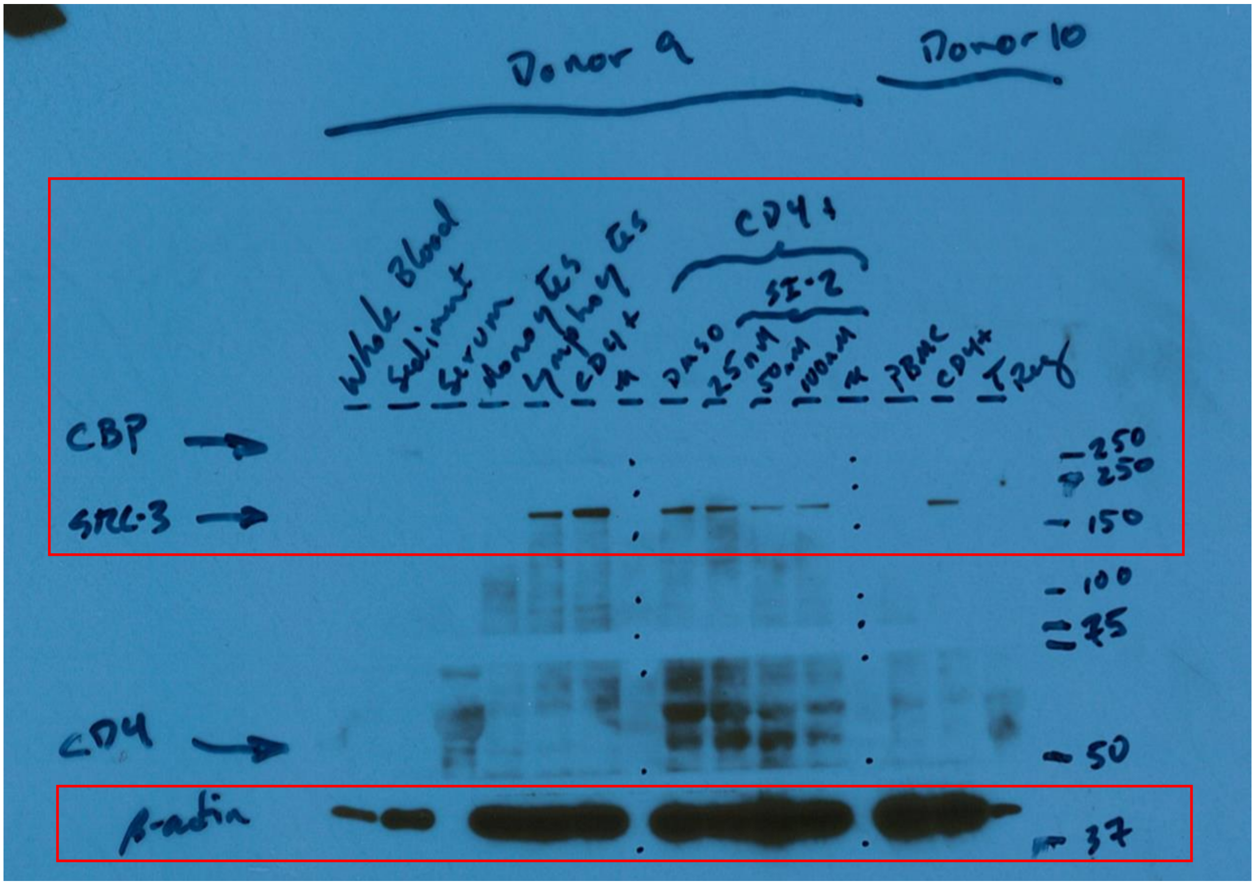

Main Figure 1D – Full gel image: Donor 10 and Donor 12

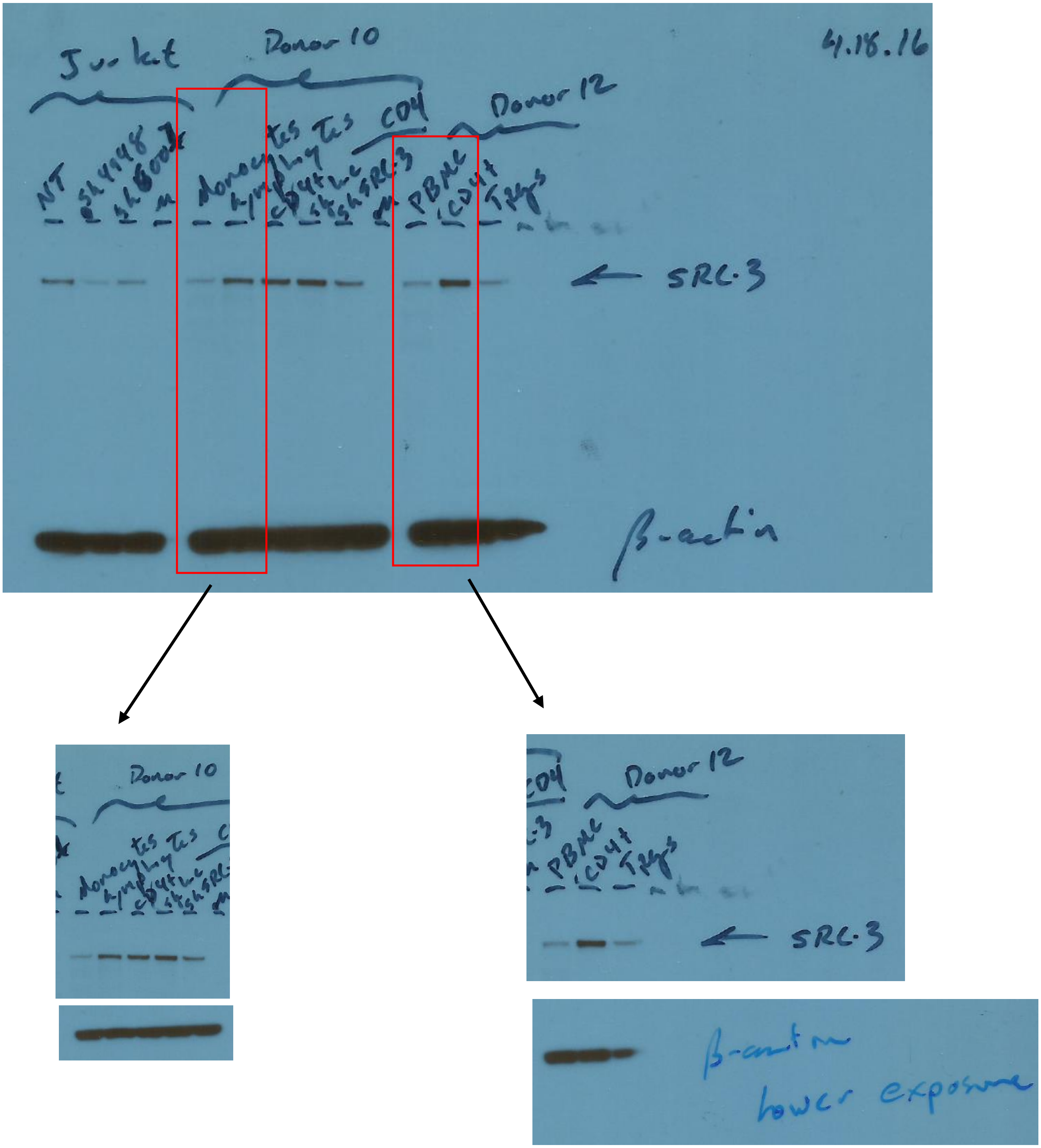

Supplement: Supplementary file 1 — Supplementary Information 1. [file 41598_2021_82945_MOESM1_ESM.pdf]
